# Static and dynamical isomerization of Cu_38_ cluster

**DOI:** 10.1038/s41598-019-44055-z

**Published:** 2019-05-20

**Authors:** Chuanchuan Zhang, Haiming Duan, Xin Lv, Biaobing Cao, Ablat Abliz, Zhaofeng Wu, Mengqiu Long

**Affiliations:** 1 College of Physics Science and Technology Xinjiang University, Urumqi, 830046 People’s Republic of China; 2 Hunan Key laboratory of Super Micro-structure and Ultrafast Process, Central South University, Changsha, 410083 People’s Republic of China

**Keywords:** Macromolecules and clusters, Condensed-matter physics

## Abstract

The lowest-energy geometrical and electronic structures of Cu_38_ cluster are investigated by density-functional calculations combined with a genetic algorithm based on a many body semi-empirical interatomic potential, the traditional FCC-truncated Octahedron (OH) and an incomplete-Mackay icosahedron (IMI) are recognized as the two lowest energy structures (energetically degenerate isomers) but with different electronic structures: a semiconductor-type with the energy-gap of 0.356 eV for the IMI and a metallic-type with negligible gap for the OH, which is in good agreement with the experimental results. The electron affinity and ionization potential of Cu_38_ are also discussed and compared with the observations of the ultraviolet photoelectron spectroscopy experiments. The dynamical isomerization of the OH-like and IMI-like structures of Cu_38_ is revealed to dominate the pre-melting stage through the investigation by the molecular dynamics annealing simulations.

## Introduction

Clusters exhibit novel physical and chemical properties and have been expected to exhibit large applications in many fields such as in catalyst, magnetism and nanoelectronics^1,2^. The size dependent properties characterize atomic cluster especially in small size range where its geometrical and electronic properties may be significantly altered with removing or adding only one atom^3^. For a given size of a cluster, its electronic structure may also be quite different for the different isomers^4,5^. Since geometrical configurations of free clusters cannot be derived directly from experimental observations, combination with theoretical calculations could be useful to identify the lowest energy geometry of a cluster from a series of candidates.

Noble-metal (Cu, Ag, and Au) clusters have attracted much attention in scientific and technological fields because of their thermodynamic, electronic, optical and catalytic properties in nano-materials^6^. Determination of cluster geometry is essential to understand its electronic properties. Structural assignment has been carried out for the small clusters of Ag_n_^+^ (n < 12)^7^ and Au_n_^−^ (n < 13)^8^ by combination with ion mobility measurements and ab initio calculations, and the critical point (size) of structural transition from planar to three dimensional is found to be considerably larger for Au_n_ than for Ag_n_^−^. And for larger clusters of Ag_n_^+^ (n = 19–79)^9^, by comparison of the experimental scattering intensity with density functional calculations, the authors conclude that those clusters studied prefer icosahedral motif. The photoelectron spectroscopy (PES) measurements on Cu_n_^−^, Ag_n_^−^, and Au_n_^−^ (n = 53–58)^10^ clusters show that the icosahedral symmetry is adopted for the Cu_n_^−^ and Ag_n_^−^ clusters but much low symmetry structures are identified for the Au_n_^−^ clusters and such conclusionare further confirmed by the density-functional theory (DFT) calculations in the case of n = 55 in the same paper, and similar conclusions are also deduced from the theoretical calculations based on semi-empirical interatomic many-body potentials^11,12^.

Many theoretical and experimental studies have shown that icosahedral-like structures with five-fold symmetry have significant advantages for small-sized clusters (containing less than 100 atoms). For example, the icosahedral geometries dominate the ground-state structures of the 13- and 55-atom clusters in most cases. However, when the number of atoms is 38, it becomes more special. It is generally believed that the ground-state of 38-atom cluster has octahedral (OH) symmetry (as like a fragment of the FCC crystal), rather than an incomplete-Mackay icosahedron (IMI). The IMI may be highly competitive at finite temperatures, which may have a significant impact on the dynamic behavior of clusters, such as the melting properties.

Experimental measurements of the ionization potentials of Cu_n_ (n < 150) clusters have been done by laser photoionization^13^, and a number of copper cluster anions have been widely studied by photoelectron spectroscopy (PES)^14–20^ to explore their electronic and structural properties. It is interesting to focus on the case of 38-atom cluster. The early PES measurements on Cu_n_^−^ (n = 6–41)^18^ give a semiconductor-type with an energy gap between the highest-occupied molecular orbital (HOMO) and the lowest-unoccupied molecular orbital (LUMO) of about 0.33 eV for Cu_38_ cluster, and the shortly performed PES measurements on Cu_n_^−^ (n = 1–411), Ag_n_^−^ (n = 1–60) and Au_n_^−^ (n = 1–233)^19^ give Cu_38_ a metallic-type with none HOMO-LUMO energy gap. Recent PES study on Na_n_^−^ and Cu_n_^−^ (n = 20–40) clusters reproduces the early PES contour^18^ of Cu_38_ cluster with a higher resolution and the authors deduce that it should not be a high symmetric cuboctahedral structure but an oblate (Mackay-type) structure of Cu_38_ cluster from analyzing the PES feature^20^.

Several theoretical investigations have been performed to explore the structural and electronic properties of Cu_38_ clusters. Most calculations based on interatomic many body potential give an cuboctahedral structure as the global minimum geometry^21–23^ of Cu_38_ except Ş. Erkoç who adopts a potential developed by himself and finds a global minimum structure with five-fold symmetry for Cu_38_ cluster^24^. Few first-principles investigations can be found of Cu_38_ cluster to our knowledge. Recently, M. Itoh and his co-workers studied the Na_n_, Cu_n_ and Ag_n_ (n = 2–75) clusters by DFT calculations with the generalized gradient approximation (GGA) of the PW91 form and an ultrasoft pseudopotential. They found the most stable Cu_38_ should be the OH structure, and with 0.143 eV higher in total energy as the IMI holds^25^. I.A. Hijazi and Y.H. Park investigated the Cu_n_ and Au_n_ (n = 21–56) clusters using an effective Monte Carlo simulated annealing method incorporated with an embedded atom method (EAM) potential, and followed by DFT calculations with PBE GGA and pseudopotential for Cu_38_, Au_38_ and Au_56_ clusters and they also denote the OH structure as the most stable one, and with 0.26 eV lower in total energy as compared with the IMI^26^.

In this study, we first adopt a genetic algorithm combined with the Gupta interatomic many-body potential to get a number of initial structures of Cu_38_ cluster, and followed by DFT calculations on these structures. The FCC-truncated octahedron (OH) and an incomplete-Mackay icosahedron (IMI) are recognized as the two lowest energy structures with only 0.014 eV difference in total binding energy. The calculated HOMO-LUMO energy gaps of the two structures are in good agreement with the experimental results. The electron affinity and ionization potential of Cu_38_ are also calculated and compared with the observations of the ultraviolet photoelectron spectroscopy experiments. To reveal the finite temperature influence as having effects on experiments, the dynamical properties of Cu_38_ with temperature are also investigated by using classical molecular dynamics simulations, and the dynamic stabilities of the IMI-like and the OH-like structures are found to be dominant in the pre-melting stage of Cu_38_.

The computational details are given in section 4, and section 2 focuses on the analysis and discussion of the results, and the conclusions are summarized in section 3.

## Results and Discussion

The global optimization of Cu_38_ is performed considering a genetic evolution group consisting fifty individuals (isomers). After 5000 iteration of evolution on the basis of the Gupta potential, the fifty individuals (structural motifs) in the final group are taken as the initial candidate structures to the DFT calculations.

**Figure 1:**
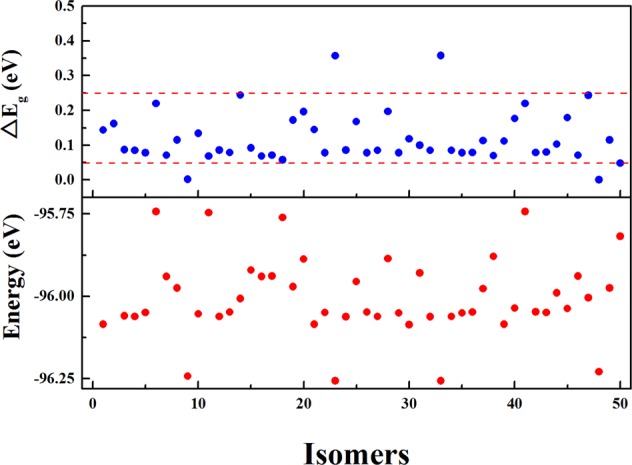
Energies and HOMO-LUMO energy gaps of the fifty isomers of Cu_38_.

Figure 1 gives the energies and HOMO-LUMO energy gaps of the fifty geometries under the DFT level (the relative energies of 50 isomers are listed in Supplementary Table S1). One should notice that after DFT relaxation two or more structures from the genetic algorithm with the Gupta potential may correspond to one structure. As show in Fig. 1, the lowest energy geometry is assigned as the IMI one, and the next nearly degenerate geometry is the OH one, and each of the two typical geometries is presented twice.

One may conclude that, at least for Cu_38_, the structural global optimization can be achieved by combing the DFT calculations with the genetic algorithm based on the Gupta potential. The genetic algorithm can afford a lot of candidate structures, by combining the first-principles calculation, which may afford an efficient way to find the lowest energy and the low-lying structures.

**Figure 2:**
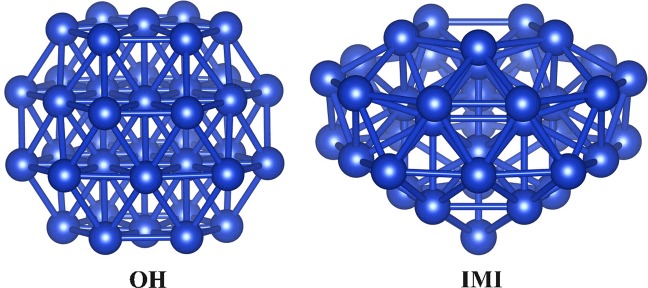
The two lowest energy geometrical structures of Cu_38_ cluster, the OH structure in left and the IMI in right.

In this study, although 50 isomers of Cu_38_ are re-optimized within the DFT calculations, Fig. 2 displays only the two isomers of the FCC-truncated Octahedron (OH) structure and an incomplete-Mackay icosahedra (IMI) structure which are the two lowest energy structures of Cu_38_ clusters. Most of the others low energy isomers are come from the distorted IMI structure, and the binding energies of these structures are all higher than that of the OH and IMI structures.

Cluster symmetries and the total binding energies of the OH and IMI structures of Cu_38_ cluster in neutral and charged (cationic and anionic) states are listed in Table 1. For the neutral Cu_38_ cluster, total binding energies of both structures are very close with the difference of 0.014 eV, and if considering the average binding energy per atom, such difference (~4 × 10^−4^ eV/atom) is too small to be considerable, the two structures can be recognized as the energetically degenerate isomers of the ground-state of Cu_38_ cluster. For the cationic cluster of Cu_38_, the total binding energy difference between the OH and IMI is also small (∼0.052 eV), and similar to the case of neutral cluster, this difference may also be negligible considering the average binding energy per atom. For the anionic Cu_38_, the energy difference (∼0.14 eV) between the OH and IMI structures is still closer to that in neutral or cationic states, and such an energy difference is still quite small (<0.01 eV/atom) if considering the relatively high temperature (for example, the room temperature).

**Table 1:** Symmetries and total binding energies of the OH and IMI structures of Cu_38_ cluster in neutral and charged states.

Isomer of Cu_38_	Symmetry	Total Binding energy (eV)		
		cationic	neutral	anionic
OH	O_h_	−91.198	−96.242	−98.520
IMI	C_5v_	−91.146	−96.256	−98.379

**Figure 3:**
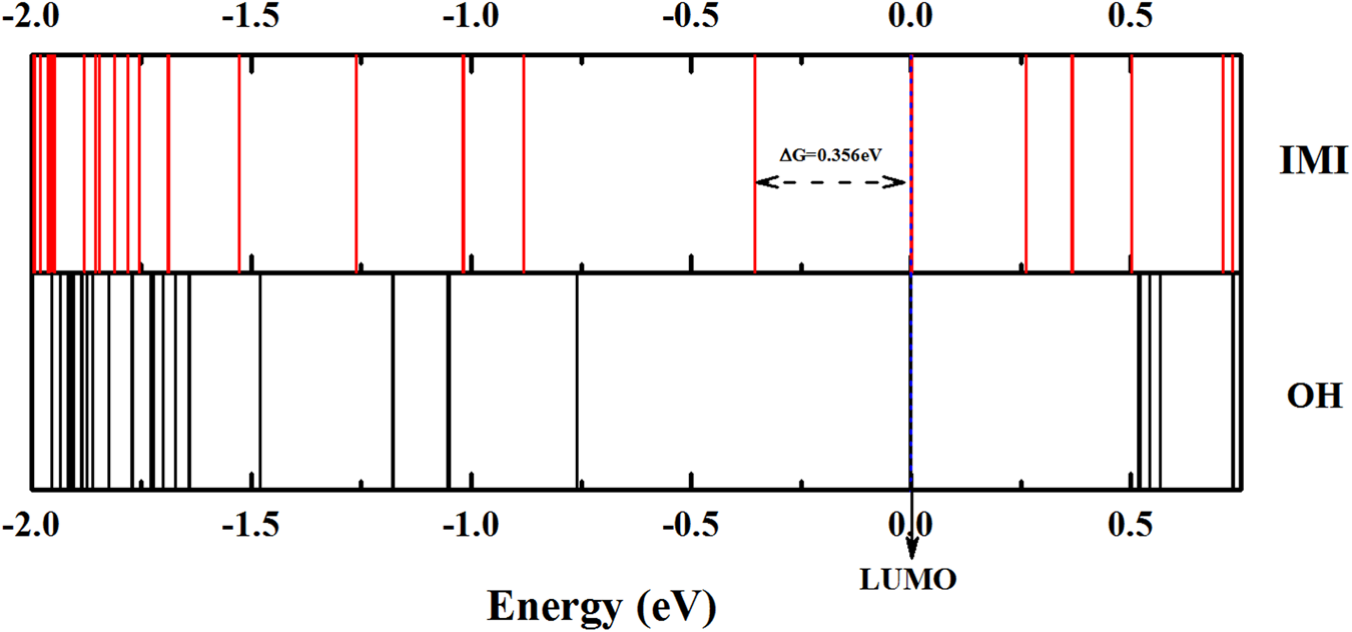
The energy levels of the IMI (upper) and OH (lower) structures of Cu_38_ cluster, and for comparison the LUMO energy lines of the two isomers are all shifted to zero as the dashed line denotes. All the energy level labels are in eV.

Although the binding energies of both structures of Cu_38_ are very close, their electronic structures are quite different. Distributions of energy levels of the two isomers (OH and IMI) of Cu_38_ cluster are presented in Fig. 3 (the IMI in top and the OH in bottom), and for comparison the LUMO energy lines of the two isomers are all shifted to a same value (in zero point) as the dashed line denotes in Fig. 3. From Fig. 3 we can see that the energy level distributions of the OH and the IMI are quite different, the significant differences are the HOMO-LUMO energy gaps, an energy gap of 0.356 eV is found for the IMI structure, while for the OH structure, the gap is too small that can be negligible.

As stated above, several experimental PES investigations on copper clusters have been done to explore the electronic structures of those clusters. A semiconductor-type with a HOMO-LUMO energy gap of about 0.33 eV of Cu_38_ cluster is found from the early PES measurements on Cu_n_^−^ (n = 6–41)^18^, and this energy gap is quite closer to the calculated value of the IMI structure (0.356 eV), and so we assign this PES feature to an IMI structure of Cu_38_ cluster. The recent PES studies on copper and sodium cluster anions by O. Kostko *et al*.^20^ give a high resolution PES picture of Cu_38_^−^ cluster but the substantial features are same with the early PES study^18^, and from analyzing the 2p shell distributions, O. Kostko *et al*. deduced that it should not be an OH structure of Cu_38_, and predicted an oblate (Mackay-type) structure of Cu_38_ cluster, and this structural assignment is also agreement with the IMI structure we emphasized above. The another PES measurements on Cu_n_^−^ (n = 1–411), Ag_n_^−^ (n = 1–60) and Au_n_^−^ (n = 1–233)^19^ give Cu_38_ cluster a metallic-type with none energy gap, and we assign this PES feature to that of the OH structure with a negligible HOMO-LUMO energy gap as shown in Fig. 3.

It is interesting to note that, though the energies of the two typical geometries are not quite different from those of the other isomers, the energy gaps of the two typical structures are distinctive. The OH has a negligible gap and the IMI has a gap of 0.356 eV, and the gaps of all the other isomers are between 0.05 to 0.25 eV (as represented by two dashed lines in Fig. 1). The energy gaps of the OH and IMI are in good agreement with the experimental observations (a zero gap for the OH and a gap of about 0.33 eV for the IMI).

Table 1 shows that all those energies are quite closer for the two isomers (OH and IMI) of Cu_38_ cluster in both neutral and charged states. If ignoring the occurrence of structural transition between the OH and IMI structures for the possibly relatively high energy barrier in the process of obtaining or losing one electron, we can obtain directly the electron affinity (EA) and ionization potential (IP) for the two isomers of Cu_38_ cluster: the EA is about 2.123 eV in IMI structure, while its 2.278 eV in OH structure. These calculated EAs are all less than the experimental results obtained from the PES investigations as stated above for Cu_38_ cluster: 2.82 eV in the previous experiment^18^ (we assign to the IMI structure) and about 3.0 eV in the subsequent experiment^19^ (we assign to the OH structure). Such underestimations (0.2–1.7 eV lower) between the theoretical (generally DFT-based methods) and experimental results about the EAs are also found in theoretical calculations of the copper clusters in small size^27,28^. From Table 1 the calculated IPs are 5.04 and 5.11 eV, for the OH and IMI structures, respectively, and these calculated IPs are closer to but all less than the experimental result (5.61 eV).

**Figure 4:**
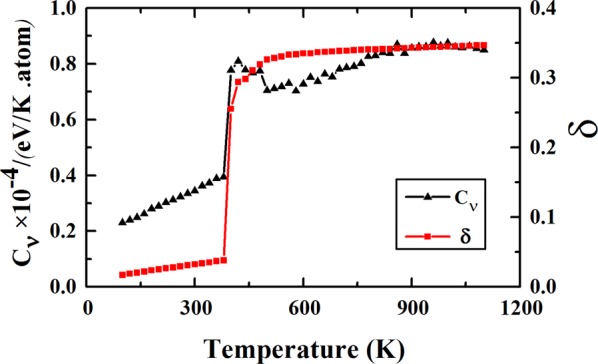
The heat capacity (C_*v*_) and the Lindemann index (δ) of Cu_38_ at different temperature.

Considering that the experiments have been done at finite temperature (e.g., the room temperature), the molecular dynamics (MD) annealing simulations are performed to reveal the dynamical properties especially the energetic and structural variations of Cu_38_ with the temperature. Many different MD annealing simulations of Cu_38_ are performed in order to satisfy the statistical convergence. The heat capacity and the Lindemann index of a typical MD annealing process are shown in Fig. 4. The heat capacity curve looks more irregular as comparing with the Lindemann index. There is no clear regular peak in the heat capacity curve which can be due to the structural competitions between the two typical series (IMI-like and OH-like) which will be discussed below. The Lindemann index shows a typical three-stage evolution: At 380 K and lower temperatures, the value and variation of Lindemann index are all quite small and Cu_38_ maintains in a solid-like state (a slightly distorted OH structure); From 380 K to nearly 520 K, the Lindemann index increases rapidly, especially an abrupt jump occurs from 380 K to 400 K which may correspond to a distinct structural variation of Cu_38_, and the temperature range (380 K–520 K) can be viewed as the temperature interval of the pre-melting stage of Cu_38_. After 520 K, variation of the Lindemann index becomes small and tends to saturation (~0.35), and Cu_38_ is totally in a liquid-like state.

**Figure 5:**
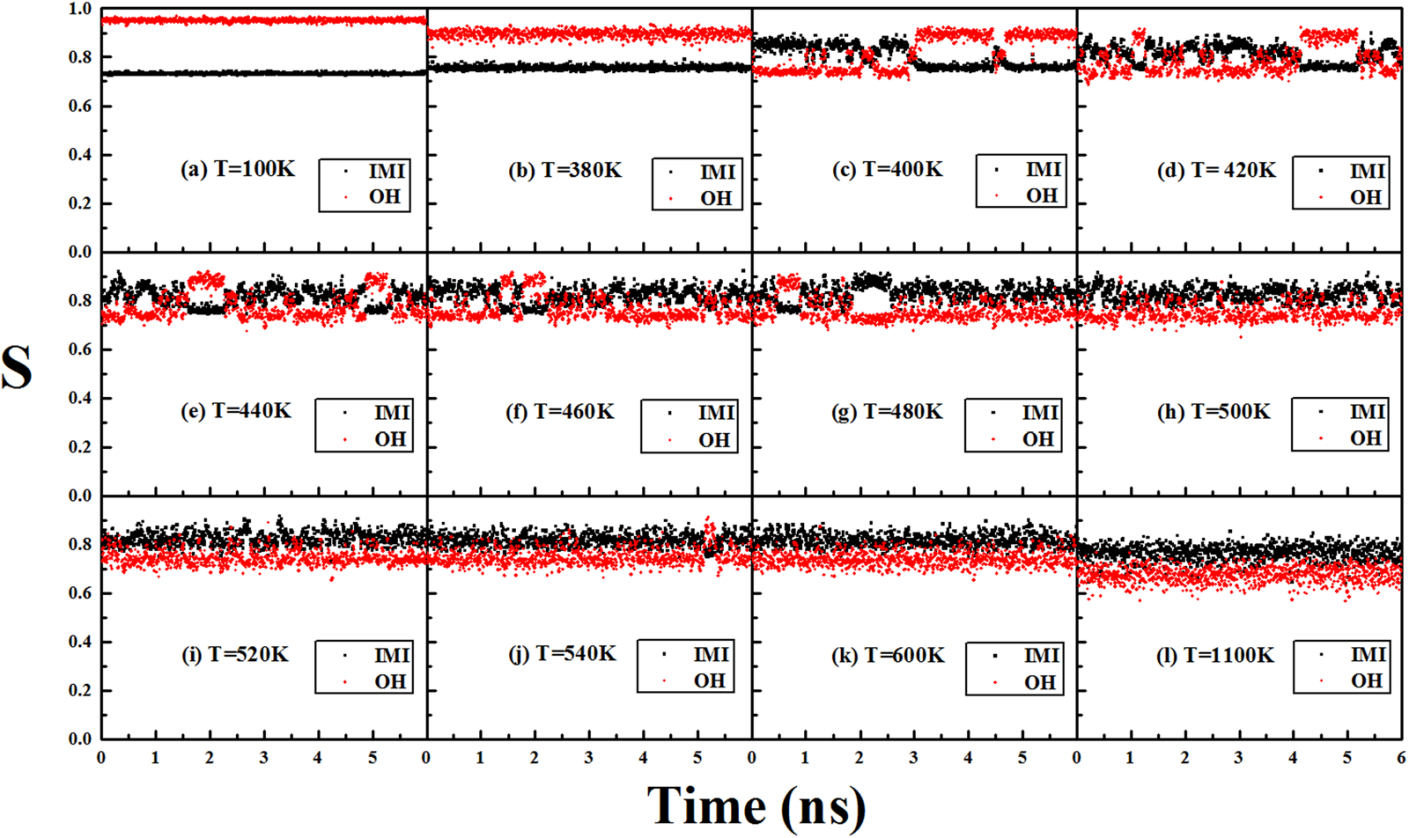
The similarity functions of between the ideal structures (OH and IMI) and the dynamical structures at different temperature of Cu_38_.

To investigate the dynamical structural variations of Cu_38_ with temperature and especially the dynamical stability of the two typical geometrical motifs: the OH structure and the IMI one, the dynamical structure of Cu_38_ at each given time and temperature can be compared to the ideal OH and IMI structures and the similarity between them can be described by the similarity function. Figure 5 gives the similarity functions between the dynamical structures of Cu_38_ and the two typical ideal structures (OH and IMI) at several different temperatures. The snapshots of Cu_38_ at different temperatures are shown in Supplementary Fig. S1.

As stated above, Cu_38_ is in a solid-like state at the temperature of 380 K and before, and all the snapshots of the dynamical structures of Cu_38_ in this temperature scope correspond to the OH structure with only slight distortions (a perfect OH structure can be obtained through relaxation of each of these structures). As shown in Fig. 5(a,b) at the temperature of 100 K and 380 K, the value of similarity function with the ideal OH geometry tends to about 0.95 and 0.9, respectively, and the values of the similarity function with the ideal IMI geometry are all less than 0.8 (around 0.75), which is consistent with the snapshots of the OH-like dynamical structures of Cu_38_ at these temperatures.

At 400 K, variation of the similarity function is substantially different from that at 380 K, and strong structural competition between the OH-like structures and the IMI-like ones begins to dominate the process. Generallythe IMI-like structures dominate the first half time and the OH-like ones dominate the rest time. The abrupt structural variation from the OH-like ones at the end of 380 K to the IMI-like ones at the beginning of 400 K and the strong structural competition between the two typical structural motifs cause the abrupt jump of the Lindemann index from 380 K to 400 K, as shown in Fig. 4

From 400 K to 520 K, as can be seen from Fig. 5(c–i), the IMI-like and OH-like structures still dominate the dynamical structural process of Cu_38_ in general, and the structural competition becomes more complicated with increasing the temperature. Such a temperature scope just corresponds to the pre-melting stage of Cu_38_, which implies that at the pre-melting stage of Cu_38_ the dynamical structural variations is mainly determined by the competition between the two typical structural motifs (the IMI and the OH). It is this strong structural competition between the OH-like geometrical motifs and the IMI-like ones of Cu_38_, causing the irregular variation of the heat capacity around the pre-melting stage, as shown in Fig. 4.

At 540 K and higher temperatures, as shown in Fig. 5(j–l), variations of the similarity functions with the two typical ideal structures become indistinguishable, and Cu_38_ can undergo many different structures and the cluster is overall melt, which is consistent with the nearly saturated Lindemann index, as shown in Fig. 4.

To address the reliability of the SA MD with the Gupta potential, the SA MD from the first-principles are also performed. For the huge DFT computational efforts, the DFT SA MD processes are performed only at four temperatures from 600 K to 300 K, and the temperature interval is 100 K, and 1 × 10^4^ MD steps are propagated with the time step of 5 fs at each temperature. Similar to the process of the SA MD with the Gupta potential, the initial geometry at the lower temperature is originated from the final structure at the neighboring higher temperature in process of the DFT MD.

**Figure 6:**
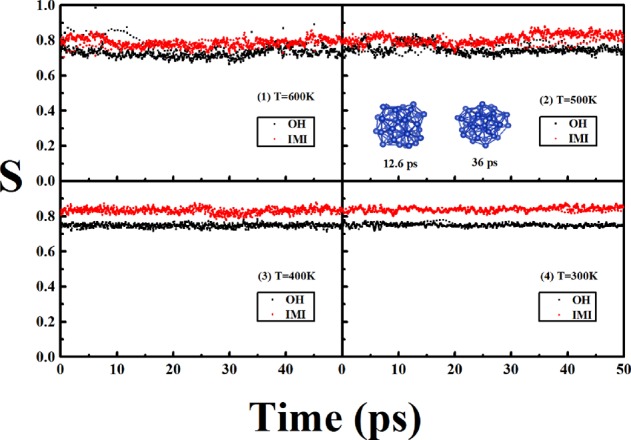
The similarity functions between the ideal structures (OH and IMI) and the dynamical structures at different temperature of Cu_38_ under the DFT level. Two snapshots at different time are shown at 500 K, as corresponding to distorted OH-like and IMI-like geometries.

Figure 6 gives the similarity functions to the OH and IMI of Cu_38_ under the DFT level, one can clearly find the distinct structural characteristics of Cu_38_ at different temperatures. The structural variations are quite complex and neither IMI nor OH is commonly ergodic at 600 K and 500 K, and Cu_38_ sustains in a liquid-like state at these higher temperatures. From 500 K to 400 K, a substantial structural transition occurs, and the structures become distinctly relatively monotonous and IMI-like geometries become dominant at 400 K, and Cu_38_ sustains in a solid-like state at 400 K and the lower temperatures.

**Figure 7:**
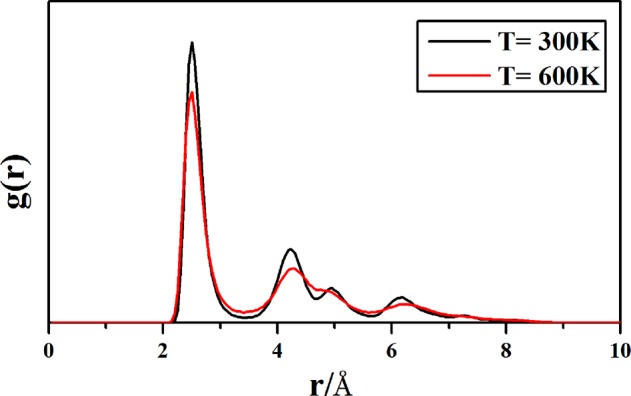
The radial distribution function (RDF) of Cu38 at 600 K and 300 K. Each RDF is obtained by averaging over 1000 structural samples.

The correlation of the phase (solid-like or liquid-like state) of Cu_38_ and the temperature is further investigated by analyzing the fluctuations of the radial distribution function (RDF). The RDF can be calculated as an ensemble average over atomic pairs: $g(r)\propto V\langle \sum _{i}\sum _{j\ne i}\delta (r-{r}_{ij})\rangle$ (where V is the volume and r_ij_ is the distance between the ith and jth atoms). Figure 7 gives the radial distribution functions (RDFs) of Cu_38_ at two different temperatures (600 K and 300 K). At 600 K, the RDF shows typical liquid-like nature: the curve changes gently and the distribution tends to be relatively uniform. The RDF is quite different at 300 K as comparing to that at 600 K: variation of the RDF becomes relatively sharp and two peaks emerge distinctively around 4 ~ 5 Å at 300 K, which reflects a solid-like characteristics of Cu_38_. From 500 K to 400 K, the RDF changes from liquid-like to solid-like as corresponding to the pre-melting stage of Cu_38_, and as an example, two snapshots (a distorted OH-like geometry and an IMI-like one) of the dynamic structures of Cu_38_ at 500 K are shown in Fig. 6.

From Fig. 6, the typical transition stage with the temperatures from the solid-like to the liquid-like of Cu_38_ can be figured out, which implies that the pre-melting stage of Cu_38_ occurs around 400 K–500 K from the first-principles calculations, which is in well agreement with results of the SA MD under the Gupta potential (as shown in Fig. 5).

## Conclusions

The static and dynamical structural properties of Cu_38_ cluster are systematically investigated through the combination of the first-principles calculations and the classical molecular dynamics simulations based on a semi-empirical many-body potential (the Gupta potential). The lowest energy geometrical and electronic structures of Cu_38_ cluster are investigated using density-functional calculations within the general gradient approximation, with a previous process of obtaining initial structures from the combination of the genetic algorithm and the Gupta potential. The high symmetric octahedron (OH) and the incomplete-Mackay icosahedron (IMI) with five-fold symmetry are recognized as the energetically degenerate isomers of the ground-state of Cu_38_ cluster, with a difference of only about 0.014 eV in total binding energy. The electronic structures of the IMI and the OH structures of Cu_38_ are substantially different and the calculated HOMO-LUMO energy gaps of the two isomers are in good agreement with the different experimental observations: the IMI shows a semiconductor nature with a finite energy gap of about 0.356 eV, and the OH shows a metallic-type with a negligible energy gap. The calculated ionization potentials and electron affinities of the two typical geometries (IMI and OH) of Cu_38_ are also closer to the experimental results. The thermal stabilities of the IMI-like and OH-like structures of Cu_38_ are revealed to play an important role at the pre-melting stage of Cu_38_, and the irregular variation of the heat capacity of Cu_38_ can be due to the complicated competition between the OH-like structures and the IMI-like ones. The above results all stress the occurrence and importance of both the static and the dynamical isomerization in the case of Cu_38_ cluster.

## Computational Methods

As the number of stable geometrical configurations (the local minima on energy surface) increase very rapidly with the cluster size, it is hardly to explore all possible configurations. In this study, a two-step method is applied to identify the global minimum structure of Cu_38_ cluster. At first, mass initial structures are generated by combination of a genetic algorithm (GA)^29^ with the Gupta-type many-body interatomic potential^30^, and then the 50 lower-energy isomers are chosen and optimized further by the density functional theory (DFT) calculations, and finally the lowest energy one among these structures within the DFT calculations is taken to be the ground state geometry (the cartesian coordinates of the 50 isomers are shown in Supplementary Table S2).

All first-principles calculations are carried out using DFT with the Dmol3 software^31,32^ in Materials Studio from Accelrys Inc. The exchange correlation interaction is treated within the general gradient approximation (GGA) using Perdew-Burke-Emzerhoff (PBE)^33^ functional. Non relativistic all electron calculations and double numerical basis with polarized functions (DNP) are adopted. The convergence threshold in self-consistent field electronic calculations is set as 10^−6^a.u on total energy. In the process of geometry optimization, the convergence criteria are set as 10^−5^ Ha for total energy, 0.005 Å for the displacement, and 0.002 Ha/Å for the forces. All clusters are fully optimized without symmetry and spin constraints.

For the finite temperature observation of the behaviors of Cu_38_ clusters, the dynamical properties of Cu_38_ with temperature may also be important. As it’s still a hard task from the first-principle dynamical simulations of Cu_38_ for the huge computational efforts, the classical molecular dynamics (MD) simulation is used to investigate the annealing behaviors of Cu_38_ from a relatively high temperature of 1100 K (the cluster is in a liquid-like state) to a relatively low temperature of 100 K (the cluster is in a solid-like state).

The semi-empirical many-body Gupta potential^33^ is used in the MD simulation. The Gupta potential can be written as the sum of a Born-Mayer type repulsive part and a many-body attractive part, as shown below:

1 $${\rm{V}}=\sum _{{i}}(\sum _{{j}(\ne {i})}{Aexp}[-{p}(\frac{{{r}}_{{ij}}}{{{r}}_{0}}-1)]-\sqrt{\sum _{{j}(\ne {i})}{{B}}^{{2}}\exp [-2{q}(\frac{{{r}}_{{ij}}}{{{r}}_{0}}-1)]})$$

Where r_0_ is the nearest-neighbor distance, and r_*ij*_ is the distance between the ith and jth atoms. The parameters of Gupta potential for copper are chosen as A = 0.0855 eV, B = 1.224 eV, p = 10.960, q = 1.867 and r_0_ = 2.56 Å^34^.

The Simulated annealing (SA) method is adopted to reveal the dynamical properties especially the structural variations of Cu_38_. The SA process of Cu_38_ is as follow: Firstly, for any chosen stable structure (for example, this can be achieved by using the stochastic method combined with the steepest descent method), it is heated directly up to 1100 K, and then it is slowly cooled down to 100 K. The intervals of time and temperature are 1 fs and 20 K separately, and 6 × 10^6^ MD steps are propagated at each temperature point.

The Lindemann index (δ) (i.e., the root-mean-square bond-length fluctuation) and the heat capacity (C_*v*_) of Cu_38_ are calculated as follows:

2 $${\rm{\delta }}=\frac{1}{{\rm{N}}}\sum _{{\rm{i}}}{{\rm{\delta }}}_{{\rm{i}}}$$

3 $${{\rm{\delta }}}_{{\rm{i}}}=\frac{1}{{\rm{N}}}\sum _{{\rm{j}}(\ne {\rm{i}})}\frac{{(\langle {{\rm{r}}}_{{\rm{ij}}}^{2}\rangle -{\langle {{\rm{r}}}_{{\rm{ij}}}\rangle }_{{\rm{T}}}^{2})}^{1/2}}{{\langle {{\rm{r}}}_{{\rm{ij}}}\rangle }_{{\rm{T}}}}$$

4 $${{\rm{C}}}_{{\rm{\nu }}}=\frac{{\langle {{\rm{E}}}_{{\rm{t}}}^{2}\rangle }_{{\rm{T}}}-{\langle {{\rm{E}}}_{{\rm{t}}}\rangle }_{{\rm{T}}}^{2}}{2{{\rm{NK}}}_{{\rm{b}}}{{\rm{T}}}^{{\rm{2}}}}$$

where k_b_ is the Boltzmann constant, and E_t_ is the total energy of the cluster. N is the total number of atoms in the cluster, and denotes the ensemble average.

The similarity function (S)^35^ between two different structures is also used to reveal the structural characteristics of Cu_38_ at different temperatures.

5 $${\rm{S}}=\frac{1}{1+{\rm{q}}}$$

6 $${\rm{q}}={[\frac{1}{{\rm{N}}}\sum _{{\rm{n}}-1}^{{\rm{N}}}{({{\rm{r}}}_{{\rm{n}}}-{{\rm{r}}}_{{\rm{n}}}^{\text{'}})}^{2}]}^{1/2}$$

7 $${{\rm{r}}}_{{\rm{n}}}=|{{\rm{R}}}_{{\rm{n}}}-{{\rm{R}}}_{0}|$$

where N is the cluster size (N = 38) and **R**_0_ is the geometrical center of the cluster (${{\bf{R}}}_{0}=\frac{1}{{\rm{N}}}\sum _{i=1}^{{\rm{N}}}{{\bf{R}}}_{{\rm{i}}}$, and **R**_i_ is the position vector of the ith atom), and r_n_ (r′_n_) is the distance from the nth atom to the geometrical center of the cluster. For a given cluster all the distances are sorted in increasing order. The more similar the two clusters are, the more closer to 1 the S value is.

## Supplementary information

Supplementary Information
