# Supplementary material for: Static and dynamical isomerization of Cu38 cluster
Source: Sci Rep. 2019 May 20;9:7564. doi: 10.1038/s41598-019-44055-z (PMC6527573; doi:10.1038/s41598-019-44055-z)
Supplement: Supplementary file 1 — Supplementary Information [file 41598_2019_44055_MOESM1_ESM.pdf]

Supplementary Information

Static and dynamical isomerization of Cu_{38} cluster

Chuanchuan Zhang, [1] Haiming Duan,[1,*] Xin Lv,[1] Biaobing Cao,[1] Ablat Abliz,[1]

Zhaofeng Wu,[1,*] Mengqiu Long[1, 2]

[1]College of Physics Science and Technology Xinjiang University, Urumqi 830046,People's Republic of China

[2]Hunan Key laboratory of Super Micro-structure and Ultrafast Process, Central South University, Changsha 410083, People's Republic of China

*Corresponding author: dhm@xju.edu.cn

*Corresponding author: wzf911@mail.ustc.edu.cn

Table S1 The isomers and the relative energies (The energy differences between the energies of the isomers and that of the lowest-energy structure) of Cu_{38} under the DFT level.

Isomers	Relative Energy (eV)	Isomers	Relative Energy (eV)
1	0.171	26	0.208
2	0.205	27	0.195
3	0.197	28	0.37
4	0.195	29	0.205
5	0.207	30	0.17
6	0.513	31	0.327
7	0.316	32	0.194
8	0.281	33	0.000
9	0.014	34	0.195
10	0.203	35	0.205
11	0.520	36	0.208
12	0.195	37	0.279
13	0.208	38	0.377
14	0.249	39	0.171
15	0.335	40	0.22
16	0.316	41	0.513
17	0.317	42	0.209
18	0.495	43	0.207
19	0.285	44	0.266
20	0.369	45	0.219
21	0.171	46	0.317
22	0.207	47	0.252
23	0.000	48	0.027
24	0.194	49	0.281
25	0.301	50	0.438

Table S2 Cartesian coordinates of the 50 isomers of Cu_{38} under the DFT level.

Isomer 1			Isomer 2		
X	Y	Z	X	Y	Z
-1.554718	-2.298236	0.200701	-2.881185	-1.504257	-1.67715
-3.784814	-1.388595	-0.261331	-2.864938	1.797247	0.504969
-2.401365	-2.339898	-2.217947	-1.603184	-0.338649	0.19386
2.397253	-3.561041	-0.571862	-0.399272	-0.90606	-1.903156
1.902607	-1.761764	-2.297114	-1.144937	-3.122029	-2.5326
1.01065	-1.606748	0.049443	1.374414	-2.813142	-2.055958
0.518624	-3.635594	1.234585	-1.8693	-2.97628	-0.015416
0.000632	-3.357831	-1.406113	0.331219	-1.639701	-4.067702
-0.117121	-2.355183	-3.699644	-1.947884	-0.361761	-3.708761
0.875116	0.067595	-3.605497	-1.89903	2.187951	-3.570802
-3.005267	0.176492	-1.93557	2.040529	-0.397608	-2.565508
-1.707406	-0.492754	-3.964454	1.58095	2.146793	-2.032331
-0.551182	-0.899832	-1.774375	-0.777767	1.568599	-1.501795
-0.94536	1.497848	-2.677464	0.220822	0.866214	-3.741181
1.16329	0.709983	-0.902294	-3.120001	0.9387	-1.810664
3.404582	-1.162046	-0.381794	0.089687	3.763675	-0.674104
1.4239	2.376455	-2.728997	-1.762831	3.981465	1.029469
3.116064	0.494428	-2.314008	-2.374128	3.402938	-1.359636
2.768174	-2.501008	1.651251	2.220589	-2.145886	2.060151
-1.327921	-2.79168	2.632674	0.398746	-0.294873	2.028223
-0.412973	-0.549826	1.668579	2.149851	-0.314773	3.816439
-2.868554	-0.932535	1.971739	-0.214022	0.484052	4.25571
-3.592954	1.047582	0.573544	-2.016155	0.534154	2.499956
-1.323449	0.831349	-0.180165	0.37819	-2.072039	3.762472
-2.709055	2.596424	-1.263347	3.416157	0.899479	-0.879327
-0.244618	3.13669	-0.986637	2.864345	-1.487404	-0.362056
2.45375	2.514776	2.117856	2.854943	0.300324	1.500011
-0.21982	3.146993	3.133807	2.054064	2.531338	0.471724
3.553586	1.224792	0.137739	0.592953	4.055671	1.812213
0.949909	-1.845235	3.186195	-1.004485	2.707659	3.236972
0.677473	0.817661	3.468585	-0.402335	1.903524	1.010482
0.411429	1.826017	1.188279	1.391319	1.916409	2.892086
2.187681	-0.054604	1.700884	1.067467	0.334444	-0.315489
0.627706	4.292122	1.106575	2.189257	-3.806378	0.205211
2.146871	3.08838	-0.413771	-0.003084	-4.412677	-0.703665
-1.249018	-0.729609	4.00305	0.426985	-2.048321	0.185511
-1.747638	3.148271	0.9991	0.166448	-3.774009	1.840874
-1.826065	1.27016	2.557802	-1.524393	-1.904791	2.170968

Isomer 3			Isomer 4		
X	Y	Z	X	Y	Z
-3.069117	2.154656	0.444257	0.740708	-3.716518	2.409031
-1.766218	0.576277	-0.860454	0.790508	-1.782012	0.922938
-0.606502	-1.638362	-1.051386	-0.308303	-3.763412	0.03354
0.24704	-0.526078	-3.183635	-0.679955	2.43733	2.920529
-2.937053	2.31865	-2.165512	0.499817	-1.540804	3.548867
-4.170248	0.408908	-1.169748	-2.700864	1.58917	1.350395
-2.332978	-0.025194	-3.106445	-1.186248	-0.290982	0.606031
-1.299732	3.651613	-0.840746	-1.417859	-2.400429	2.00401
2.507417	2.944626	1.16302	-2.424192	2.710614	-1.00089
0.473736	2.56249	2.857639	-3.394067	-0.868157	1.482192
-0.500603	1.902944	-2.624846	0.662184	0.436322	2.176504
3.127718	1.123554	-0.526474	-1.597347	-0.028164	3.071244
1.979801	1.313893	-2.825747	-1.798472	3.953208	1.06773
-0.396047	2.036923	0.67111	-2.793093	1.207454	-3.114102
0.484127	4.329759	0.914935	-1.21025	-3.523434	-2.275783
2.498969	-0.993678	-1.91636	-2.427335	-2.270122	-0.414703
0.729555	0.486936	-0.905824	-3.230116	0.188638	-0.823331
1.25243	2.966458	-1.060277	-2.16271	-1.167032	-2.798466
-1.7129	3.679323	1.875948	-0.221168	-1.501081	-1.362604
0.323017	-2.394718	2.438429	3.357899	0.887736	-1.246004
0.269212	0.006674	3.450947	1.165326	0.328871	-0.340424
2.582624	-3.283661	1.634391	-0.308348	0.420959	-3.85726
2.555747	-1.081651	2.743091	1.283964	1.950056	-2.299177
3.830523	-1.158196	0.474187	1.794261	-0.576045	-2.631071
1.597697	0.749551	1.502846	-0.917565	2.8326	-3.127686
0.874252	-2.995391	-2.341987	0.100696	-2.025634	-3.765916
-1.471469	-2.34614	-3.266263	2.267999	-3.705329	0.452674
-0.61642	-0.42179	1.136607	2.921794	-1.470428	-0.433244
-3.152207	-0.438897	1.001184	1.463755	-3.122256	-1.836955
-1.794404	-2.674533	0.917338	2.748386	-1.880279	2.275364
-3.029306	-1.787464	-1.242979	3.071908	0.490536	1.283134
0.49051	-3.741555	0.171198	-0.923194	0.89362	-1.592663
-1.502386	-3.882101	-1.312329	-0.020902	3.836192	-1.029218
-1.888816	-1.272247	3.042602	2.103159	2.659754	0.039623
2.845539	-3.208976	-0.830138	1.972236	2.407569	2.575997
1.478732	-1.524633	0.352845	2.47364	0.16599	3.721708
3.958166	0.944075	1.860025	-0.344833	2.079304	0.556727
-1.860406	1.237959	2.578551	0.648581	4.156195	1.451258

Isomer 5			Isomer 6		
X	Y	Z	X	Y	Z
-3.038149	-1.507081	0.408595	1.444314	2.957861	0.723297
-2.9359	-1.037727	2.85043	-2.015400	-0.196517	2.568756
-2.900277	-1.753689	-2.024978	-0.280984	1.451770	1.595106
-2.950935	0.847552	1.220414	-1.829962	2.287711	3.276595
-1.473262	0.115145	-0.989591	-0.550513	-1.807411	3.969330
-2.237423	1.69212	-2.758336	0.037164	0.683604	3.977412
-2.55119	2.640599	-0.417791	1.618820	0.502513	-0.027580
-3.837524	0.473792	-1.119791	0.575857	3.090543	3.127793
0.197602	1.402443	-3.572401	2.083686	1.061546	2.423535
-0.396381	2.87517	-1.589248	1.776748	-3.662998	-1.814249
0.528544	2.760809	2.5593	0.696417	-2.032794	-0.190505
-0.491472	1.610952	0.719437	2.972236	-1.590563	-1.049800
-0.550413	4.106158	0.559923	2.768520	-1.272668	1.437193
-2.128548	3.055089	2.087252	-1.394095	-2.498294	1.684697
-1.405675	1.022431	3.237164	2.547269	-3.606208	0.497623
-1.388409	-0.52172	-3.518426	1.899233	-1.138881	3.842567
1.96454	2.901082	-2.286217	0.053931	-4.278433	0.188258
0.938511	0.773892	-1.341646	0.988921	-3.131304	2.342188
1.579469	2.848491	0.20319	0.423982	-0.804118	1.977604
0.934892	-2.194655	2.289238	-0.345673	-2.739354	-2.525904
2.656215	-2.507427	0.553116	0.287616	-0.249287	-2.002945
3.051465	-0.962599	2.690456	-2.206336	-1.074029	-2.264423
2.906673	1.553741	1.906069	1.773285	-1.634062	-3.350942
-0.861464	-0.674351	1.442822	-0.542190	-0.883406	-4.211716
0.96108	0.343258	3.101505	-0.395278	3.282558	-2.990573
0.507402	-3.868826	0.509677	2.741086	0.460166	-2.350969
-1.708094	-3.369072	-0.509224	1.673111	2.572362	-1.750430
1.648828	-0.150307	0.785655	-0.534929	1.907917	-0.859236
-0.730352	-1.348197	3.894189	0.874794	1.245995	-3.835153
-1.455533	-2.98122	1.93982	-1.567129	1.171024	-3.189686
1.637622	-3.240037	-1.742282	0.053927	4.362476	-0.851591
2.810892	-1.023598	-1.403727	-1.140843	3.678470	1.189082
4.202147	-0.45266	0.606355	-2.697699	1.667159	0.981209
0.975359	-1.08274	-3.087408	-1.893377	-2.854756	-0.622820
3.32026	1.381936	-0.746428	-3.087462	0.977031	-1.271699
0.257551	-1.654555	-0.597342	-2.350068	3.405839	-1.107641
-0.722651	-2.79896	-2.684211	-1.123589	-0.344075	0.028041
2.684601	0.724758	-3.175564	-3.335391	-0.967389	0.437580

Isomer 7			Isomer 8		
X	Y	Z	X	Y	Z
-2.420887	1.407995	1.599399	-2.935749	2.821942	0.008467
-3.668663	-0.733534	0.774008	-2.758348	0.726723	-1.347679
-2.669963	-0.474601	3.147531	-2.182358	2.897554	-2.376565
-1.287931	-0.805774	0.922067	-0.765453	3.979338	-0.61791
-0.65617	0.882896	3.648792	2.10999	1.981651	1.703252
-0.357074	-1.542517	3.168934	0.198681	1.722119	3.441039
-2.387897	-2.691232	2.15168	0.210155	2.261093	-2.182438
1.956788	-2.492141	2.745871	2.74366	2.052284	-2.315626
1.670068	-0.079902	3.07969	1.372057	0.27432	-3.237504
0.429884	-3.630682	-1.044969	-0.722223	1.646523	0.198229
-0.042768	-3.256743	1.371177	0.162216	3.502974	1.669851
1.142966	-1.069172	0.850841	1.451606	0.696803	-0.558302
2.349453	-3.399163	0.509295	1.641248	3.23032	-0.367477
-1.920602	-2.914956	-0.245984	-1.974624	2.370945	2.277333
3.524115	-1.140031	0.979074	0.355585	-0.752306	4.190765
2.698224	1.248289	1.240613	2.473246	0.242185	3.401109
-0.857529	3.169471	2.667969	3.338037	-0.45811	-1.552008
0.126875	1.241672	1.341205	0.596752	0.000028	1.782853
1.439854	2.343527	3.218116	3.760116	1.692459	-0.128856
-2.673178	-0.932871	-1.551755	-1.721419	0.015573	2.975909
-1.146565	1.028303	-0.897201	0.756357	-4.056108	-1.002118
-2.609425	0.975854	-3.092271	2.858075	-2.671474	-0.109984
-1.892509	3.095287	-2.106335	1.469058	-2.000502	-2.230824
-3.495436	1.395395	-0.698689	-1.439198	-3.349784	0.415973
-0.133262	1.59586	-3.160423	0.661952	-1.829268	0.115152
0.536758	3.235675	-1.390983	3.074196	-0.409027	1.00299
1.117635	3.473128	1.017542	0.836429	-3.983081	1.421692
-1.333658	3.216746	0.276208	1.908832	-2.033363	2.451574
-0.128735	-1.247643	-1.328569	-0.623623	-2.2272	2.470309
0.908254	-2.557971	-3.247801	-1.083818	-2.688997	-1.915545
1.589326	-0.221766	-3.093551	-0.306528	-1.433878	-3.947986
-0.889332	-0.730572	-3.613104	-1.282291	0.934104	-3.651439
-1.487338	-2.950513	-2.644612	-2.621948	-1.10073	-3.048956
3.629523	0.453756	-1.069407	-0.498124	-0.300371	-1.701513
2.393631	-1.746136	-1.297043	-3.065303	-1.890032	1.851064
2.325393	2.095659	-2.792715	-3.185573	-1.746627	-0.716193
1.286597	0.861999	-0.870682	-3.381897	0.591618	1.099437
2.93358	2.896407	-0.563917	-1.429773	-0.709693	0.531924

Isomer 9			Isomer 10		
X	Y	Z	X	Y	Z
-0.934773	1.539823	0.04428	-3.353007	-1.244916	-2.084841
-0.494019	3.885675	-0.717006	-1.949828	2.721327	2.835615
-3.621615	-0.158825	1.643803	-2.787954	2.011022	0.573709
-2.554237	2.657511	-1.50574	-2.664973	-0.421966	0.224434
-3.104397	0.309937	-0.77764	-3.025634	0.219921	2.631382
-1.364226	-0.851336	0.810281	-0.915288	-0.890209	-1.50201
-1.14024	3.423492	1.680938	-1.607084	-2.779246	-2.955465
2.338429	-0.764789	3.134516	-0.921903	0.846186	1.671171
-1.633887	1.10571	2.537623	-0.999603	0.859533	4.16561
0.058245	-0.080103	3.981034	-1.055058	-1.406477	3.077995
0.481047	2.290933	3.220474	-2.950992	-2.261346	1.83182
-3.200881	2.194243	0.891876	1.217702	-2.467499	2.753329
2.760593	1.606684	2.376242	1.085812	-0.015724	3.098159
1.177524	2.86278	0.868592	0.157043	-3.127498	-1.115167
0.713767	0.384827	1.608796	-0.680047	-3.450655	1.395603
-1.985922	-1.298864	3.197293	0.07689	-1.244956	0.819382
0.29372	-2.065649	2.447767	2.01868	-2.942222	0.44862
-1.767586	-3.206409	1.556558	-2.227365	-2.812787	-0.468555
-3.401205	-2.066279	0.002808	4.014907	-1.598869	-0.509094
-1.177541	-2.863085	-0.868468	2.547111	-2.835718	-2.030443
-2.760418	-1.606788	-2.376236	2.845959	-0.90774	1.582318
-0.713806	-0.384929	-1.60885	3.702516	0.799555	-0.057949
-2.33868	0.764836	-3.134786	0.662208	2.447763	2.778689
-0.480794	-2.291193	-3.220071	1.337396	0.953547	0.749343
1.140616	-3.423361	-1.680297	3.030432	1.537064	2.334594
0.493425	-3.885706	0.717458	1.97855	3.255737	0.605763
3.40114	2.066279	-0.002838	1.600823	-0.78086	-1.019986
0.934764	-1.539866	-0.044411	0.662669	-1.80983	-3.243169
3.621677	0.15903	-1.64403	-1.562882	-0.218427	-3.716455
3.104657	-0.309841	0.777538	-0.968342	2.27154	-3.15717
3.201462	-2.19367	-0.892124	3.264344	-0.190904	-2.560009
2.554056	-2.657375	1.505311	2.102109	1.86851	-1.528806
-0.293906	2.065547	-2.447803	-0.494023	1.41799	-0.820794
-0.05844	0.079848	-3.981145	0.768277	0.584737	-3.049994
1.364329	0.85109	-0.810329	-2.748823	1.117547	-1.837573
1.634039	-1.105678	-2.537683	0.443962	3.655883	-1.46238
1.9857	1.298964	-3.197276	-0.569475	3.350645	0.805623
1.76738	3.206536	-1.556455	-2.035107	3.48934	-1.263305

Isomer 11			Isomer 12		
X	Y	Z	X	Y	Z
-0.316187	-1.197034	-3.265173	0.813579	-1.861449	-0.190728
-2.403210	-2.827758	1.203061	-0.455949	-3.809131	-0.726865
-1.779250	-2.241998	-1.307106	2.233176	-3.555959	-1.199124
-1.028106	-0.933363	0.680585	-0.715592	-2.661012	1.687139
3.769507	0.092361	-2.115063	-2.452159	-2.197917	-0.23011
1.938784	-3.006669	1.629628	-2.78698	-1.226046	2.084071
-3.598061	-0.938631	0.028153	-1.945541	-2.97009	-2.54524
0.720670	-1.707036	-0.861418	-3.357547	0.235136	0.037607
3.204227	-1.519424	-0.272437	-1.409327	1.518815	-1.041718
1.722446	-1.220294	3.680173	-1.185857	-1.235392	-4.148976
2.082722	-3.701002	-0.813960	-1.552353	1.089987	-3.410196
-0.329448	-2.276681	2.625311	0.847605	0.07718	-3.322838
-0.183328	0.330530	3.122956	-3.610734	1.783576	-1.950545
-2.545855	-0.447524	2.445293	-3.183941	-0.66215	-2.383911
-0.210273	-3.647913	0.354177	-2.944726	2.745462	0.335338
2.156614	-1.761511	-2.774216	-1.001426	-0.350311	0.67684
1.384196	-0.697646	1.330475	-0.898069	-0.878612	-1.752261
0.115164	-3.526523	-2.356640	0.61429	-2.495555	-2.767246
3.658120	-0.903326	2.118552	-0.589283	3.805855	-0.357099
3.763105	1.040131	0.480052	2.371233	1.970849	2.174166
1.800385	0.553571	-0.798748	3.093651	-1.974076	0.664592
0.049459	1.303095	0.834096	0.033283	1.995477	3.431435
1.932075	2.861651	0.536187	0.43555	2.471545	-2.383428
-0.890675	3.428627	0.330223	3.282629	0.578935	0.109204
2.293787	1.223712	2.593171	2.73611	1.403841	-2.233351
-1.968873	2.072504	2.374402	1.58231	-2.128476	2.847973
0.417594	2.863075	2.617266	1.681035	0.20647	3.976263
3.004936	2.449633	-1.726375	-0.057385	1.914132	1.043763
0.490047	2.609146	-1.685951	3.572213	-0.341109	2.463893
-0.575862	0.373413	-1.386315	0.960952	3.988364	1.778332
1.546116	0.698643	-3.253936	1.327239	-0.132684	1.55338
-3.411000	3.278177	0.390631	2.612045	-1.099384	-1.781695
-2.113725	2.513218	-1.757514	0.922709	0.556655	-0.828083
-0.844996	1.307317	-3.577041	1.920003	2.809829	-0.265478
-2.751716	-0.047245	-2.250049	1.386061	-3.893922	1.113623
-4.452641	1.324057	-0.977820	-0.584777	-0.537482	3.181835
-2.342552	1.114697	0.191455	-1.476391	3.631417	2.10551
-4.304197	1.164015	1.613915	-2.217634	1.227234	2.253929

Isomer 13			Isomer 14		
X	Y	Z	X	Y	Z
-1.647604	-2.607923	2.95064	-1.129849	-3.198492	-2.738917
-0.310713	-0.897684	1.570497	3.018469	-1.329837	-0.887508
0.284955	-3.394967	1.442106	-0.317404	-1.016222	-1.858697
2.265052	2.281181	1.786384	2.434512	-3.75058	-0.301963
3.653789	-1.163398	-0.200306	-2.344477	-0.933784	-3.122577
-0.171882	2.444369	2.914625	1.350406	-2.768109	-2.318757
3.975093	1.411787	0.042404	0.015959	-1.542471	-4.204839
2.733654	0.590727	-1.953862	-0.212423	-3.976285	-0.548042
0.584367	-1.93606	3.660518	0.856188	-1.958101	0.152915
1.258071	0.374961	2.984615	2.80865	-2.053217	1.651856
-0.158924	1.714899	0.632724	-1.047569	3.262897	-2.887363
3.609019	0.134511	2.111593	1.77976	-0.213924	-2.908833
0.814383	4.076565	0.98007	1.220264	2.262976	-2.253006
1.752506	0.246	0.374181	-0.916441	1.374365	-1.363811
1.915336	2.592212	-0.716544	-0.477867	0.852432	-3.72852
2.151066	-1.81296	1.739792	-2.81947	1.513086	-2.961512
-1.219373	-0.004662	3.601644	-0.154833	3.798498	-0.624228
-1.591611	3.711995	1.087492	1.32017	0.448162	-0.563232
-2.292196	1.431348	1.836566	2.117024	2.67781	0.17396
0.390014	-1.587154	-0.700128	3.231969	0.45987	1.01754
-0.224361	-3.871189	-1.077816	1.013583	-3.865221	1.741
2.167795	-3.273812	-0.266022	1.881332	2.003144	2.700415
-1.857836	-2.694398	0.451677	0.990808	-0.146745	1.893159
-3.037871	-0.921622	1.851089	2.865475	-0.308457	3.430005
-1.902655	-2.052713	-1.957056	-1.724002	3.756416	1.384933
-3.875674	-1.591419	-0.511315	-0.702077	2.259019	3.245909
-0.324988	3.465003	-1.24468	0.646207	0.432415	4.287615
-1.197639	-0.360418	-3.606865	-2.568607	1.413278	1.37642
-1.467399	2.047026	-3.02531	-0.109198	1.913349	0.957259
1.246959	-0.182256	-3.737453	-1.324738	-0.275739	2.93948
-2.367499	2.192473	-0.71683	-2.200917	-2.261406	-0.694718
-3.146179	0.272562	-2.184983	-3.110276	0.188568	-0.870883
-4.111309	0.739268	0.236581	-2.649848	2.912447	-0.779874
-1.78614	-0.190414	-0.219794	0.804894	-2.000182	3.391107
0.102877	0.726293	-1.61991	-1.01632	-0.341886	0.435313
0.21893	-2.385272	-3.136094	-3.142219	-1.065589	1.342597
1.11373	2.313107	-3.100416	0.713125	4.025725	1.833138
2.454253	-1.83797	-2.279819	-1.100262	-2.548204	1.662658

Isomer 15			Isomer 16		
X	Y	Z	X	Y	Z
-0.407391	-2.176192	-3.510335	-3.624577	-1.216458	0.172123
1.612415	0.490009	-0.447862	-3.009624	-0.842509	2.796437
3.293833	-1.228166	-1.242612	0.637509	1.326837	3.928618
-1.560456	1.736218	-2.739811	-3.759818	1.043061	1.429222
-0.37343	-0.453103	-1.787939	-1.652919	1.309481	2.839389
2.618383	-3.256116	0.185384	-0.502023	1.732643	-0.293432
1.728562	-0.61181	-3.113288	0.458631	3.141371	-2.072824
-2.413736	-0.643688	-3.260174	-0.88066	-0.496626	-4.069156
0.883862	1.790491	-2.490013	-1.418468	1.535878	-2.806078
1.338184	-2.674977	-1.83108	0.987909	1.015098	-3.255131
-0.281896	0.266534	-4.23775	-0.174062	4.228204	0.081502
0.768438	-1.634844	0.378801	1.854185	2.633458	0.21942
3.078947	-1.008275	1.250843	-2.915377	1.070932	-0.87099
3.270823	1.137379	-2.33869	-2.879436	-1.024341	-2.304098
4.118839	0.870325	-0.072285	0.174776	2.826942	2.04657
-0.794776	1.565691	-0.31592	-2.199213	2.92901	1.030684
2.561267	1.404683	1.728062	-1.543331	-0.267324	0.848302
-0.093083	3.696757	-1.095087	-2.02597	3.437753	-1.405722
2.320194	2.795297	-0.592857	-0.669694	-0.495415	-1.574116
-0.986637	-2.884132	-1.074812	0.679161	0.428887	1.587532
0.042245	-3.847827	1.006681	-1.027258	-2.691607	-2.998714
-0.461578	-1.916056	2.612809	1.519927	0.328038	-0.864195
-2.329266	-2.817052	1.111567	2.789716	-1.807284	-1.076768
1.831514	-2.845676	2.487558	1.20782	-1.465766	-2.986706
1.768056	-0.515258	3.314636	2.307651	-3.491107	0.619196
-3.989258	-0.752809	0.80167	3.244709	0.008218	-2.657311
-3.146195	0.564987	-1.222461	0.803085	-3.410552	-1.486515
-2.366376	-0.28329	2.646501	0.501768	-1.734544	0.292069
-3.200799	-1.925131	-1.247187	2.767642	-1.038213	1.291474
-1.579635	-0.683061	0.348119	2.799556	2.326192	-2.022367
-2.722724	3.037537	-1.02053	3.726048	0.841774	-0.114228
0.6699	2.955242	1.289734	2.762241	1.39628	2.236158
-0.502677	0.077111	4.197419	-1.703246	-2.662882	-0.643068
-1.336668	2.083139	2.863517	-0.099355	-4.170242	0.713122
-1.619395	3.729635	1.028566	-0.545705	-0.862237	3.405086
-2.955539	1.565776	1.055541	-1.532022	-2.549995	1.83911
1.049954	1.943399	3.592029	0.944503	-2.6563	2.534982
0.166094	0.447254	1.74126	1.995921	-0.676655	3.590423

Isomer 17			Isomer 18		
X	Y	Z	X	Y	Z
-0.93343	2.841072	-2.324748	-0.385925	-1.849105	-4.080157
3.131062	2.528963	-1.185362	0.974805	0.359261	-0.729525
1.246193	0.892783	-0.906931	2.799779	-1.392819	-1.073039
1.445792	2.553409	-2.957217	-1.462739	3.373608	-1.991975
1.982711	-1.812921	3.269682	-0.504633	-1.356795	-1.668188
3.449415	0.53162	0.195897	1.477969	-0.445836	-3.060417
2.253537	2.485473	1.200314	-2.593243	-1.070584	-2.815917
0.048996	-0.091082	3.214818	0.719727	2.146006	-2.353185
-3.273587	2.305404	-1.363502	-0.658319	0.583002	-3.860498
-1.555468	3.119296	0.165211	3.05059	1.059044	-1.771461
0.997843	2.166283	3.406448	3.142046	0.401942	0.72003
3.661636	-1.559513	1.432303	-0.473729	2.185246	0.113988
-0.037026	1.388273	1.112702	2.14827	2.277778	2.517496
2.668792	0.50376	2.648693	0.497568	4.291808	-0.902015
0.803293	3.384201	-0.629965	2.061868	2.564356	-0.015009
0.152831	3.797413	1.789148	-1.61737	0.959555	-1.736827
-1.528511	1.963043	2.884889	-3.249731	2.445399	-0.611198
2.664589	-1.534716	-0.954597	0.676831	4.011561	1.516645
1.279127	-0.768022	1.064039	-1.847703	3.914256	0.904424
2.698012	0.306365	-2.703023	-1.489863	-3.429257	-2.510417
1.243341	-1.791459	-3.127161	2.243938	-3.686693	-0.228402
-0.762185	-3.427897	-2.298693	1.165895	-2.958368	-2.35458
-1.277284	0.754709	-1.058991	-0.330965	-3.740568	-0.3381
-2.369084	1.070262	-3.33987	0.823472	-1.769965	0.490009
-3.925299	-1.070108	0.887117	2.959254	-2.009531	1.588905
0.141509	0.489633	-3.190765	0.999814	-3.751124	1.91899
-1.648567	-1.20424	-2.835896	0.800169	0.470105	1.717579
-3.535397	-0.177025	-1.394053	2.865584	-0.083575	3.148158
-1.284168	-0.868824	0.879987	-0.648921	2.297165	2.674328
-2.512397	-2.89495	1.697565	0.74276	0.776122	4.136803
-0.233231	-2.458405	2.485484	-2.696277	1.434448	1.535404
-2.499613	-2.358829	-0.787428	-1.316944	-0.193027	3.022039
1.795867	-3.176305	1.070572	-2.491425	-2.338194	-0.433807
-0.527246	-3.510137	0.235747	-3.548288	-0.040996	-0.5739
-2.44978	-0.457284	2.919296	0.845709	-1.597176	3.141094
1.478693	-3.648782	-1.340239	-1.227298	-0.285257	0.47068
0.094876	-1.420203	-1.068221	-3.254123	-1.068033	1.666674
-2.885842	1.148745	0.906752	-1.198554	-2.483762	1.825373

Isomer 19			Isomer 20		
X	Y	Z	X	Y	Z
3.248422	0.850518	-1.387775	0.969216	-1.542047	-0.117884
1.084141	0.284394	-0.463123	2.100412	-3.434417	-1.377778
-0.567165	0.551141	-3.709772	0.970825	0.303434	-3.481657
1.179727	1.976673	-2.353391	0.509313	-2.113469	-2.855803
-2.927638	1.092659	-2.616693	3.02197	2.162555	1.608075
1.664807	-0.569815	-2.787173	3.201773	-2.05375	0.567075
-1.051279	2.92515	-3.145946	-1.113072	1.341378	2.702693
-0.106127	-1.998558	-3.944333	1.113136	2.875544	-2.332309
-1.559712	-3.169308	-2.029014	3.346314	0.389182	-0.08542
-2.420164	-1.197494	-3.489971	3.090599	1.24327	-2.561693
-0.607478	-0.994393	-1.791239	1.089751	-1.788457	2.40008
-1.876882	-2.734998	0.540002	0.948531	0.327459	3.739539
0.284231	-3.49376	2.078768	1.027148	3.031134	0.245397
2.154941	-3.855106	0.20806	3.078159	-0.268375	2.346379
-0.220502	-4.397978	-0.309304	0.918721	2.727815	2.770028
2.833343	-1.548381	-0.644246	1.019355	0.639371	1.33545
1.091864	-3.019142	-2.017501	2.623668	-1.100531	-1.962657
2.529088	-2.080821	1.912859	1.086501	0.782663	-1.10921
3.076908	0.396096	1.110334	3.217231	2.851955	-0.769284
0.512817	-2.06709	0.181621	1.377449	-3.814445	0.944935
-1.26303	-1.477947	2.738341	-1.056257	1.453368	0.073203
0.915445	-1.880708	3.885491	-1.031011	3.565028	-1.119753
-1.297359	-0.339412	0.554814	-3.076108	-1.990224	2.041117
-1.008894	1.421437	-1.24028	-3.108952	1.970278	-1.194828
-0.15826	3.697743	-0.826802	-3.352991	-0.038346	0.329419
2.053918	2.576162	0.054587	-1.089012	-0.75656	1.128389
1.752829	2.070164	2.64855	-1.042337	3.563935	1.466882
0.81376	-0.313002	1.952255	-3.271065	0.264522	2.869135
2.606396	-0.077053	3.573076	-3.106388	2.210216	1.337664
-1.927618	3.407832	1.13038	-1.146067	-1.12416	3.518947
-0.583189	2.87676	3.295693	-1.291407	-0.592726	-3.909419
0.056172	0.571331	4.034507	-1.076806	1.531589	-2.587996
-3.100989	1.285018	0.1339	-0.90918	-3.131842	1.565417
-0.128489	1.842614	1.073103	-0.396497	-3.474058	-0.90105
-2.019346	1.003947	2.49	-2.547722	-2.311799	-0.338921
-3.010818	-1.028083	-1.072585	-3.176863	-0.363456	-2.180172
-2.644682	3.300568	-1.267516	-1.971023	-2.632184	-2.709358
0.620815	4.112836	1.500328	-0.947314	-0.70385	-1.394631

Isomer 21			Isomer 22		
X	Y	Z	X	Y	Z
-0.197994	1.958165	-3.525444	-0.12858	2.441666	-3.396597
0.854817	2.28844	-1.334056	1.706222	2.207575	-1.685416
1.686764	-0.022976	-0.642319	1.687756	-0.05581	-0.712413
3.73991	0.416499	0.554959	3.569056	1.314937	-0.13651
3.298501	1.837972	-1.486284	3.232033	0.457615	-2.611821
1.309386	-3.453399	1.784393	1.972462	-2.912567	2.335789
1.796809	0.602407	-3.064256	0.835825	0.185306	-3.192785
1.665034	-3.980665	-1.069493	2.046067	-2.592666	-0.136065
3.789748	-0.561153	-1.854845	3.962341	-1.226918	-1.012055
-1.457298	3.317425	-1.739149	-0.304558	3.499851	-1.077859
2.961465	-2.093688	-0.024355	3.300978	-0.925882	1.322273
2.804982	1.283922	2.669047	2.266892	1.204587	2.012493
2.569384	2.830486	0.650761	0.772664	-0.905466	1.367059
-0.646846	-0.49424	-1.34652	2.068336	-0.778058	3.55364
1.636733	-1.798397	-2.359749	1.805753	3.30264	0.577622
-0.490956	-2.985294	-2.194168	-0.724319	4.008576	1.276364
0.454664	-2.167043	0.015996	-1.806943	2.025934	2.507334
-0.520532	-4.444934	0.000595	0.005502	0.535038	3.260867
-0.321634	-0.614154	-3.722062	0.126952	1.614412	0.858242
1.85657	-0.89541	1.848806	0.677208	2.901046	2.931043
0.516144	0.674455	3.513326	-2.595839	-1.68386	-2.231247
-1.917007	-0.118318	3.40996	-0.081	-3.272404	0.973353
-2.118548	-2.500768	-0.374775	-0.651339	1.009404	-1.358418
0.116025	-1.732125	3.337671	-2.358439	2.581356	-2.366206
-0.948691	-0.510859	1.228789	-1.578479	-0.174358	0.799522
-3.21978	-1.438447	1.639642	-2.025021	-3.346686	-0.51633
-1.269595	-3.030087	1.872104	1.969282	-1.888367	-2.494504
-2.48041	-1.521919	-2.686335	-3.143925	0.505118	-1.236477
-1.358893	1.547666	-0.141923	-3.63818	-1.519415	0.050613
-2.226261	0.991978	-2.625758	-0.410766	-1.948081	-3.391748
-0.025385	3.596135	0.574016	-2.298906	-0.380466	3.172819
-2.521953	3.506764	0.764011	-0.331278	-1.462829	-0.951103
-1.20344	2.145777	2.439997	-0.27688	-1.986937	3.278139
-3.222677	1.087659	1.483544	-2.250361	-2.478265	1.834023
0.778602	1.308725	1.137855	0.292764	-3.683054	-1.595376
-3.141118	-0.277673	-0.60307	-2.267358	2.403306	0.118336
-3.665241	2.134892	-0.931502	-3.736825	0.724664	1.313747
1.118716	3.112184	2.800593	-1.689097	0.299057	-3.44034

Isomer 23			Isomer 24		
X	Y	Z	X	Y	Z
3.773696	-0.285987	0.200963	1.169052	1.543935	3.385578
0.388750	4.061451	4.928310	-1.307753	2.376404	3.556836
0.393816	-0.970917	-1.993420	-0.664418	1.818625	-1.006068
1.248418	-0.288767	2.799059	-0.310953	3.677754	-2.522831
1.243748	2.184646	-0.605502	-0.04346	3.867669	0.10326
2.469328	2.840959	5.632192	2.018672	2.581267	1.12442
0.388750	4.061452	-1.928311	-0.286837	1.763163	1.478453
0.395092	-2.586461	2.764843	-2.297381	3.058019	1.285275
-0.887670	-0.786059	1.500000	-2.359508	3.60777	-1.116879
-0.892419	3.346936	0.154763	0.466841	-0.618339	2.012766
3.770262	2.187352	3.605066	1.26289	0.500548	-0.071743
-0.892421	3.346935	2.845238	3.085325	-1.277078	-0.239894
-0.889175	0.789167	-0.673568	2.393678	0.151047	-2.317573
-0.889176	0.789169	3.673569	2.201362	-2.325869	2.005174
2.527752	0.370163	-1.978560	1.716678	2.556699	-1.465756
2.523370	4.460036	-0.651659	3.584159	1.220547	-0.343382
1.243747	2.184647	3.605503	2.853258	0.232701	1.890976
3.770264	2.187351	-0.605067	-0.802408	-0.062654	3.949937
3.769294	3.717355	1.500000	1.840542	-0.740994	4.027035
2.477046	-2.011578	4.049915	-3.513285	-1.026612	0.467252
0.387096	5.547848	0.120007	-1.772307	-2.678645	-0.392479
2.469327	2.840960	-2.632191	-2.512916	0.655385	2.143142
2.477046	-2.011577	-1.049914	-0.814858	-2.528426	-2.839502
4.983383	1.507049	1.500001	-3.105713	1.310825	-0.391886
2.465933	5.842943	1.500000	-1.211315	-0.272963	0.207932
2.530989	1.504656	1.500000	-1.394384	-0.327815	-4.079596
0.393816	-0.970915	4.993422	-2.178769	1.691625	-2.850157
2.532243	-2.159450	1.500000	-2.578529	-0.717359	-1.894586
2.523370	4.460036	3.651658	0.999978	-0.981039	-4.119696
0.390101	1.435110	-2.777653	0.39555	1.476136	-3.452198
0.387096	5.547846	2.879994	-0.054392	-0.418261	-2.002895
0.395092	-2.586461	0.235158	0.652304	-1.926506	-0.12779
2.527752	0.370165	4.978557	0.124119	-4.112159	-1.176141
0.390100	1.435110	5.777654	1.773271	-2.393808	-2.2398
1.248418	-0.288767	0.200941	0.131525	-2.487227	3.592221
1.242065	3.713089	1.500000	2.309827	-3.702974	-0.066205
3.773694	-0.285988	2.799037	-1.759547	-1.843671	2.100247
-0.049994	1.500493	1.500001	-0.010299	-3.64772	1.386555

Isomer 25			Isomer 26		
X	Y	Z	X	Y	Z
1.292296	0.420587	-1.022199	-3.861158	-0.740464	0.308127
3.246009	0.524555	0.757715	-1.293065	-0.56123	-3.67141
3.451444	2.262489	-0.889931	-1.765531	1.919967	-3.06723
1.656013	-3.293474	1.94598	0.667996	1.048826	-3.478265
2.186866	1.806414	-2.931277	-3.53228	1.062941	-1.396795
1.495973	-3.973777	-0.410298	-2.99749	-1.258137	-1.997332
3.469286	-0.19441	-1.759617	-1.43545	-0.268041	1.013206
3.009051	-1.887902	0.109156	-1.05601	0.376061	-1.379392
2.581041	1.362208	3.052208	-1.081407	-2.80175	-2.399621
2.317757	3.123214	1.205417	2.728611	-1.544884	-0.928721
1.596003	-2.040636	-2.057174	0.93125	-1.327368	-2.882168
0.605485	-1.739651	0.220864	1.764864	-3.14675	0.681816
-0.710581	-3.584325	1.173337	3.035034	-0.010275	-2.876805
1.852889	-0.895275	2.261681	1.408246	-3.495908	-1.818768
0.219938	0.440569	3.624592	0.088216	-1.720921	-0.560196
-0.173563	-2.097319	3.249791	-2.260455	-2.560212	-0.037617
0.829947	1.214666	1.311167	-0.398163	-4.191454	-0.301583
0.573293	2.885211	3.152526	-0.522811	-2.76298	1.736702
0.867707	-0.183191	-3.532167	-1.950796	2.747278	-0.581258
-0.474626	-2.227742	-3.672125	-2.729894	-1.724491	2.407061
-1.632501	1.782523	2.258297	-3.324891	1.532361	1.064869
1.064658	2.9422	-0.962289	-2.243722	0.713471	3.151474
-1.023575	1.296842	-0.314784	-0.453174	-1.06209	3.514176
-1.343056	3.622632	-1.280072	2.068765	-0.207927	3.625332
-2.53144	-2.300169	2.388479	0.975041	-0.63037	1.417392
-2.792439	2.040769	-2.446599	1.608405	-2.526806	3.039908
-3.284072	0.039839	1.320317	0.167691	1.276088	3.043403
-0.946261	-0.517702	1.540791	0.068785	1.714406	0.583426
-0.359939	3.472287	0.967299	1.114282	3.384128	2.193989
-2.156136	-0.248411	3.689727	-1.484498	2.914362	2.021769
-3.131223	2.305157	0.132346	1.392137	0.523925	-1.064348
-1.841908	0.021164	-3.645696	3.690864	0.805555	-0.45289
-0.332478	1.793403	-2.705734	0.235354	2.77205	-1.775789
-0.663987	-3.301546	-1.401986	3.322679	-1.107632	1.477764
-2.299532	-1.991552	-0.015656	2.621949	2.34306	-2.416147
-3.161741	0.027564	-1.18855	2.462681	1.317152	1.683862
-2.695811	-2.100953	-2.481107	-0.108919	4.214755	0.227608
-0.760789	-0.80626	-1.644428	2.146864	2.983303	-0.105553

Isomer 27			Isomer 28		
X	Y	Z	X	Y	Z
1.447076	-3.478155	-0.249736	-3.63634	0.91948	-2.250812
-0.601631	-0.742897	0.969384	-2.535631	1.930321	-0.347392
-1.478593	-2.070134	2.826795	-0.752894	-2.599769	1.685915
0.203413	-0.229167	3.321639	0.747151	-4.084116	0.31903
-0.540768	2.289247	2.949966	-3.024121	-2.345085	0.658058
2.867664	0.838642	-1.238076	-4.046972	-0.123632	-0.035565
3.12911	-1.82408	-1.457683	-2.381294	-1.092289	2.862982
4.234754	-0.42019	0.438016	-2.624942	-1.34768	-1.638843
0.940264	-2.428077	2.107925	-3.296812	1.145282	1.94524
3.3036	-2.608847	1.162805	-1.023014	1.041124	2.861154
3.269902	1.73632	1.201728	0.041353	-1.169595	3.684283
2.688026	-0.495206	2.533785	-1.631472	-0.308375	0.691682
0.943731	1.197343	1.366439	2.533372	-0.577086	3.296586
1.804636	-0.901726	0.298943	0.767346	-0.337587	1.534596
1.923559	1.630958	3.548142	1.786624	-2.504484	1.902268
-0.623839	3.841091	0.796923	1.084985	1.204901	4.034542
1.472984	2.891396	-0.458712	-0.071996	1.72497	0.508436
-2.818175	2.785863	2.068045	0.496487	3.095998	2.544547
1.549323	3.515086	1.975839	-1.818653	3.139735	1.67108
-2.251113	0.348886	2.540815	-0.508853	-1.787394	-2.907116
-0.809125	2.843721	-1.658661	1.645644	0.233748	-0.771949
0.345043	0.676658	-1.004119	3.983183	-0.54278	-1.404937
1.253527	1.992362	-2.842205	-1.485633	2.362867	-2.666657
-2.993839	-1.505706	0.864163	-0.778074	0.357974	-1.507366
-3.722298	0.976736	0.368694	2.598957	-2.476096	-0.505627
-1.044233	-3.216571	0.588055	1.94838	-1.335445	-2.693581
-2.897594	3.359913	-0.347991	-1.925858	0.05234	-3.721781
-1.392791	1.597661	0.507795	0.95789	2.426456	-1.847289
-3.020162	1.653322	-2.122336	0.952233	-3.589759	-2.086286
-2.50633	-2.592212	-1.479271	0.509088	0.574336	-3.545055
0.000257	-1.777485	-1.22098	0.216714	-1.741837	-0.500048
-3.071565	-0.68544	-3.178068	3.280288	-0.551356	0.95302
-0.565336	-4.087125	-1.768453	2.826117	1.085396	-2.851843
-0.897568	-1.936498	-3.421163	3.387605	1.835488	-0.46043
-0.915435	0.640933	-3.175752	2.336135	1.596906	1.835935
-2.047218	-0.262713	-1.053324	-0.794676	3.744096	-0.625509
1.406644	-0.532616	-2.938851	1.639415	3.493093	0.310811
1.4141	-3.021294	-2.820514	-1.401734	-3.450143	-0.932079

Isomer 29			Isomer 30		
X	Y	Z	X	Y	Z
-0.455639	0.296274	1.753394	3.12847	-0.073542	0.237703
-2.655922	0.560043	2.667627	2.234862	1.579092	-1.451873
-1.763845	-1.97174	2.548792	2.660998	-0.773567	-2.251523
0.77899	-1.847764	2.625801	0.779928	-0.738203	-0.689832
1.582425	0.372632	3.321298	3.707676	-2.337446	-0.370942
1.663571	-0.220373	0.585644	1.508836	-2.998106	0.577247
-0.67152	-0.519393	4.184601	-0.458339	-3.143432	-1.04835
-2.400691	-3.386419	0.523263	1.896754	-3.110085	-1.852566
-3.044412	-0.957663	0.699309	1.122687	1.35532	0.781252
-0.501832	-1.699166	0.373966	3.286379	2.466136	0.63822
-0.229419	-2.337756	-3.154967	0.880784	-2.902959	3.023451
-2.083448	-2.064183	-1.52837	1.589638	3.179817	2.366437
3.583032	-1.598577	-0.434265	2.720114	0.833203	2.594378
-0.231495	-3.711192	-0.968477	0.168626	1.220329	3.155341
3.188265	-1.285306	2.093445	1.545965	-0.757303	4.157848
-0.26363	-3.881726	1.634473	-0.900523	-1.004767	3.400253
1.874325	-2.739632	-1.884589	1.011369	-0.846542	1.789971
1.751216	-2.8724	0.574631	3.178434	-1.796793	2.130161
-3.855496	-0.329742	-1.581527	-0.875709	-2.430646	1.238899
1.12094	2.391152	1.985121	-2.907822	-2.581875	-0.24301
-0.599107	1.864824	3.693296	-2.9492	-1.391502	2.075863
-1.490298	2.514354	1.449391	-2.108883	-2.629051	-2.818045
0.359659	1.748011	-0.308002	-3.625791	-0.12131	0.145346
2.47049	2.938945	-0.096193	-2.395524	1.098113	2.739882
0.193231	4.14549	0.526634	-0.508225	1.495801	-1.102032
3.316873	1.275657	1.691856	0.765208	3.250091	-2.526316
0.704955	3.732429	-1.843407	-1.73289	-0.165719	-3.592006
0.511302	-0.526679	-1.619133	-1.304898	2.298225	-3.340404
-1.612729	0.414812	-0.767954	0.685186	0.712068	-3.176927
1.922865	1.611024	-2.316837	1.063015	3.617601	-0.131477
3.649817	0.837594	-0.735249	-2.947538	1.293399	-1.851895
2.874965	-0.623994	-2.627527	-3.871775	-1.005308	-2.180484
0.738944	-0.065865	-3.957321	-0.751816	2.880872	1.473436
-1.6523	3.147733	-0.929412	-2.762719	2.156204	0.423076
-1.761917	-0.310145	-3.230167	-1.135138	0.167652	0.968523
-0.524999	1.761196	-2.745622	-1.351116	3.718069	-1.023743
-3.471807	1.54784	0.233895	-1.588468	-0.821245	-1.258624
-3.015357	1.789705	-2.437415	0.241446	-1.692591	-3.007241

Isomer 31			Isomer 32		
X	Y	Z	X	Y	Z
0.818859	-1.336293	-0.869152	1.57328	0.801658	-1.278384
0.640629	-3.990396	-1.137957	4.074131	-0.153687	1.425577
2.775794	-2.868436	-1.357235	3.11049	2.556727	-1.943105
-1.31335	-2.605008	-1.551848	0.579109	-2.792782	1.519731
-1.793314	2.954492	-1.431111	1.007333	-3.431189	-0.912355
-1.414676	-0.785795	-3.272385	3.834037	0.062856	-1.047132
-1.064522	1.756872	-3.631431	2.668255	-1.885142	0.268845
0.885071	0.341173	-3.044635	2.857481	1.332691	2.999184
-3.148101	1.120871	-2.55027	3.100712	2.08745	0.505436
-3.057215	-0.873705	-1.154955	2.060962	-1.54032	-2.21018
-0.942807	0.576104	-1.320977	0.324058	-1.149696	-0.398133
0.78463	-2.214637	-3.073015	-1.385256	-2.988028	-0.21485
0.103711	3.515709	0.104177	2.266489	-1.305395	2.884408
3.189329	-1.072982	0.344913	0.575327	0.608414	3.629238
0.618439	2.769798	-2.153373	-1.823657	-0.316335	3.955332
4.044038	1.122921	-0.430011	0.054215	-1.937718	3.812488
2.885061	1.901677	-2.485404	-0.623029	-0.564768	1.857246
2.947686	-0.532818	-2.069117	-1.977579	-2.483218	2.280144
1.481322	1.079149	-0.519995	1.648012	0.259954	1.183681
2.55898	3.205603	-0.266072	0.913125	2.568484	1.921788
3.198003	0.841823	1.911018	-3.112884	0.86331	-2.187447
2.618217	-1.448182	2.803812	-2.898911	1.822322	0.227384
1.329409	2.346975	1.849411	-3.68132	-2.303379	0.464435
-1.321664	-0.155735	3.006277	-4.473221	-0.10365	-0.367892
0.191519	-2.161783	2.842478	-3.037631	-1.736203	-2.014676
1.660143	-2.94059	0.952215	-3.245445	-0.178453	1.916487
0.970691	-0.343365	1.39625	-2.079481	-0.539264	-0.193107
-2.237764	-2.480242	2.596881	-0.41491	1.282958	0.181957
-0.732191	-3.594487	0.885453	-1.505116	1.72107	2.446877
-3.05502	-2.89115	0.268023	-0.532018	-2.147187	-2.533637
-2.160864	3.41264	1.078426	-1.338125	2.732563	-1.654505
-3.465373	-0.625826	1.418952	-0.747601	0.387431	-2.161633
-0.76001	2.317572	3.212927	-1.930224	-0.619645	-3.986425
1.071217	0.532794	3.614239	0.778868	2.155816	-3.113657
-1.232881	-1.168283	0.615107	0.626849	-0.373286	-3.974145
-3.17918	1.380125	-0.045414	2.835426	0.549616	-3.385092
-0.854031	1.347162	0.98605	-1.101887	3.563916	0.733656
-3.039789	1.566253	2.477748	1.020137	3.192112	-0.63754

Isomer 33			Isomer 34		
X	Y	Z	X	Y	Z
3.772517	-0.285435	0.201393	-3.033255	-0.693239	1.273147
0.389971	4.062134	4.929962	-1.421573	-2.22657	2.575311
0.396062	-0.971928	-1.994558	-0.562633	-0.750873	0.83652
1.247000	-0.288741	2.799146	-1.854904	-2.831499	0.163757
1.243357	2.184733	-0.605740	-3.603135	-1.469189	-1.102084
2.471038	2.841215	5.632104	-2.201697	-3.094575	-2.318477
0.389971	4.062137	-1.929959	2.428385	-3.546452	-0.431193
0.394102	-2.586937	2.765145	1.865	-2.095047	-2.525999
-0.888274	-0.786487	1.500001	1.627684	-1.212022	-0.274205
-0.891841	3.347305	0.153427	0.528355	-3.030613	1.129816
3.768836	2.187109	3.604136	0.122299	-3.728261	-1.364446
-0.891836	3.347300	2.846571	-0.282236	-2.424282	-3.747889
-0.887569	0.788799	-0.675210	0.496441	-0.02871	-3.374834
-0.887568	0.788798	3.675211	-2.115489	0.397689	-0.863428
2.529606	0.369064	-1.979825	-2.125977	-0.617156	-3.170485
2.522841	4.460344	-0.651283	-4.392018	0.814584	-0.238253
1.243354	2.184731	3.605740	-0.539571	-1.440146	-1.545586
3.768843	2.187106	-0.604135	-1.221196	1.809174	-2.685006
3.768460	3.717665	1.500003	0.375176	0.838629	-1.004016
2.477580	-2.012610	4.048504	3.91799	-1.632593	-0.967295
0.386552	5.548228	0.120221	-2.683334	2.737552	-0.708848
2.471033	2.841217	-2.632099	1.266197	2.322648	-2.731569
2.477582	-2.012613	-1.048503	2.683805	0.363527	-1.952527
4.982059	1.507454	1.500003	3.676152	0.366333	0.474293
2.465410	5.843103	1.499998	-0.900082	1.711089	0.955785
2.529298	1.504666	1.499998	3.067833	2.348411	2.033619
0.396064	-0.971925	4.994561	-0.144418	3.321267	-0.852622
2.528164	-2.154741	1.499999	2.274293	2.426372	-0.392283
2.522840	4.460349	3.651283	0.813612	3.316302	1.607931
0.392774	1.434497	-2.779558	-1.62884	4.034677	1.107283
0.386555	5.548223	2.879778	2.981561	-1.968509	1.548817
0.394101	-2.586935	0.234857	1.404138	0.748725	1.275567
2.529606	0.369064	4.979817	3.059646	0.052826	2.98621
0.392772	1.434495	5.779560	-1.186622	2.745693	3.182421
1.246996	-0.288741	0.200854	0.931798	1.463728	3.502027
1.241242	3.712433	1.499998	-3.158107	1.858081	1.698136
3.772516	-0.285438	2.798607	-1.33639	0.266379	3.006059
-0.052018	1.500361	1.499998	0.871113	-1.153951	2.894345

Isomer 35			Isomer 36		
X	Y	Z	X	Y	Z
0.862339	0.426422	-3.119227	-1.990496	-2.263385	-1.260196
-1.418554	-0.484509	-3.356565	0.533088	-2.526336	-2.07016
-3.709498	-1.149227	1.059795	-1.260977	-1.73223	-3.591922
-1.859077	-2.784219	-2.650037	-0.680707	-0.218256	-1.688425
-2.889196	-0.920131	-1.323029	-0.478207	-4.128945	-0.497642
-1.91346	-2.482938	2.37424	0.336067	-3.802667	1.799917
-1.408366	-0.495932	0.963486	3.804887	1.041409	0.03116
-2.220686	-2.953527	-0.107819	0.132517	-1.782289	0.284294
-0.004109	-3.167425	0.948452	3.538875	-1.378925	0.35177
1.879149	-2.534056	-0.832374	3.200568	0.174022	2.299799
-0.109618	-3.935913	-1.408433	2.002751	-3.280295	-0.138257
0.727286	-2.085131	-3.09832	1.900465	-1.892791	2.11946
-0.337244	-1.473871	-1.039257	1.605615	-0.022634	-0.764364
2.371551	-3.061599	1.581465	-1.033445	0.640032	-4.093445
1.074247	-0.916464	1.131936	1.139812	-0.584397	-3.463266
0.508192	-2.085521	3.189753	0.757175	1.728687	-2.714665
2.979829	-0.762766	-2.240783	2.887209	-1.744829	-2.021595
3.482482	-1.104124	0.377146	3.10016	0.81021	-2.43949
2.882148	-1.050481	2.899734	2.08726	2.583289	-0.788283
-1.86224	3.123684	-1.232743	-3.661624	-1.015298	0.148945
-1.036584	2.037625	-3.503664	-2.968598	-1.336566	2.762483
-3.148557	1.25343	-2.514642	1.157854	-0.061783	3.854678
-0.991344	0.850813	-1.253403	-3.573499	0.888216	1.979205
0.044583	3.925714	0.318285	-1.344023	0.553011	3.401398
0.583472	2.720651	-1.780697	-0.282139	1.757822	-0.338073
4.094402	1.065317	-0.570677	0.143325	3.888961	-1.634057
2.804441	1.861714	-2.477535	-1.716124	2.22997	-2.366275
1.586755	0.509532	-0.762633	0.424524	3.698427	0.951272
2.332152	2.867455	-0.052386	2.449799	2.418418	1.644723
2.736564	1.033431	1.539559	-2.922971	1.402476	-0.379892
1.335237	2.975383	2.360003	-2.977819	-0.14427	-2.364429
-1.283457	-0.393158	3.568764	0.457488	2.329354	3.137091
-2.056402	2.963591	1.2468	-1.579263	2.340862	1.721044
-0.917517	2.069818	3.229531	-1.562288	-0.173762	0.941791
0.981333	0.523803	3.231517	-1.912721	3.710961	-0.296456
-3.201318	1.186522	-0.026749	0.756477	0.437513	1.547662
0.089787	1.513097	0.930309	-0.483631	-1.673971	2.803366
-2.98872	0.93299	2.400199	-1.987386	-2.870018	1.130835

Isomer 37			Isomer 38		
X	Y	Z	X	Y	Z
3.875286	-0.81584	-1.134569	1.160784	2.556850	2.587508
1.861281	-2.024237	-1.930258	1.279904	-0.616460	1.065914
-0.474015	-3.117191	-2.213836	3.221584	2.263837	-1.531213
-3.39118	0.043657	-1.21487	2.199248	0.237989	3.098570
-0.443563	0.213977	-1.743011	3.918186	-0.597944	1.344420
-1.616604	-0.150389	0.411401	-0.321709	0.502588	3.172821
-3.737522	-1.043688	1.133212	-0.129816	1.554709	0.846786
-1.673521	-2.400725	1.696995	-2.880533	0.769571	2.325319
-2.448701	-2.293632	-0.726123	0.197839	-1.913334	2.877387
0.755069	-3.12166	1.851232	0.929731	2.440871	-2.746667
-0.966775	-4.156458	0.114909	1.304585	0.659334	-1.097894
1.426411	-3.937681	-0.449413	2.554150	-2.363260	2.352766
0.067842	-1.86749	-0.206931	2.716917	1.495794	0.885117
0.30126	-1.227889	-3.677459	1.331556	3.186254	-0.309734
2.214757	0.225458	-2.872483	-1.412468	2.682996	2.888230
0.1513	1.273287	-3.893022	-0.537020	4.030895	1.041913
-2.083383	-1.293086	-2.954719	-2.474665	2.479968	0.568747
-0.886807	3.237822	-2.746548	-0.963809	3.162111	-1.304377
-2.087412	2.185804	-0.863483	1.934820	-1.555431	-2.427659
-2.274342	1.198609	-3.158519	3.622560	-0.159572	-1.071850
-0.044563	1.726615	0.508583	-1.145442	-3.954264	-0.743746
-0.028221	3.949303	-0.497513	0.073793	-0.102285	-3.223330
1.646605	0.171206	-0.392361	-2.481789	-1.899211	-0.824136
-3.110022	1.437844	1.3896	1.151060	-3.718429	-1.565333
2.247975	2.687564	0.439283	-3.659356	-1.542661	1.445905
-1.818642	3.552604	1.217552	-0.893279	-2.420550	-2.823276
3.50609	0.406753	1.147068	2.555040	0.625083	-3.307535
1.202139	2.262929	-1.826871	-2.493741	-0.383898	-2.860687
3.596716	1.601658	-1.258636	-1.208726	0.647269	-1.131169
-2.063738	-0.33492	3.013211	2.724817	-2.293137	-0.169631
-0.961194	1.839193	2.798948	0.065929	-1.584126	-0.802345
-0.321526	-2.051084	3.781345	0.723587	-3.347132	0.896657
0.283162	0.257159	4.226055	-1.364280	1.869608	-3.479976
0.561143	3.666862	2.000598	-3.453768	0.289102	-0.143543
1.660706	1.434979	2.508491	-3.169767	2.052076	-1.833220
2.551992	-1.926523	0.646217	-1.668390	-3.051641	1.500710
2.252085	-1.087911	3.016048	-2.107380	-1.347136	3.404949
0.269912	-0.522875	1.85988	-1.300150	-0.656433	1.093602

Isomer 39			Isomer 40		
X	Y	Z	X	Y	Z
-1.676702	-2.837312	2.202893	1.77632	3.142099	1.028765
-3.012352	-1.559363	0.239855	-2.550836	-0.601625	2.839378
-1.196373	-0.540458	1.709568	-0.09416	1.493969	1.057131
-0.863803	-2.62237	-0.180421	-2.065892	1.886935	2.516389
-3.468418	-0.79564	2.556059	-0.537906	-2.167716	3.032547
-2.088115	-1.640655	-2.174357	-0.298107	0.308636	3.328959
-3.201542	0.959896	0.713218	3.591528	1.417263	0.483344
-1.169493	0.234883	-0.619468	0.360916	2.692497	3.228782
-2.488559	0.387806	-3.55025	2.048666	1.078654	2.405056
-2.431236	2.129181	-1.54108	3.698634	-0.652571	-0.974138
-3.791426	0.030086	-1.496091	3.300362	-0.900783	1.5437
-0.334928	1.230326	-2.881407	2.254961	-2.472589	-0.091095
-0.119914	2.960191	-1.049855	1.760028	-1.014289	3.697436
-0.261324	3.015681	3.036112	1.870525	-3.004009	2.29707
0.215147	1.497733	1.114487	0.810641	-0.915387	1.363818
0.646037	4.036088	1.039557	0.096379	3.247507	-0.817121
-1.530346	-0.956312	4.113533	1.54241	0.502991	-0.567443
-1.746995	2.974395	0.871153	2.550836	0.601625	-2.839378
-2.099953	1.367668	2.928296	-0.733319	3.904349	1.42068
0.416411	-1.212192	-1.80869	2.441239	2.664736	-1.393926
1.926944	-2.40319	-3.273106	0.537906	2.167715	-3.032547
1.926793	0.125861	-3.314971	0.298107	-0.308636	-3.32896
-0.394928	-1.067064	-4.048132	-2.048666	-1.078654	-2.405056
-0.310848	-3.365259	-2.587376	-0.096379	-3.247507	0.817121
3.187155	0.5579	0.643583	-0.810641	0.915387	-1.363818
3.666676	1.255779	-1.732367	-1.870525	3.004009	-2.29707
2.933801	-1.128611	-1.222489	-1.54241	-0.502991	0.567443
1.589805	-3.180494	-0.827449	-1.77632	-3.142099	-1.028765
0.716215	-3.565862	1.442608	2.065892	-1.886935	-2.516389
2.923836	-2.187385	1.178008	-3.300362	0.900783	-1.5437
2.461501	-0.263848	2.777809	-3.591528	-1.417263	-0.483344
0.983056	-0.931329	0.658112	-0.360916	-2.692497	-3.228782
0.575042	-1.823701	3.083658	0.094161	-1.493969	-1.057131
2.162772	2.245953	2.346013	-2.441238	-2.664736	1.393926
1.782863	2.657033	-2.663086	0.733319	-3.904349	-1.42068
0.307782	0.652984	3.484503	-2.254961	2.472589	0.091095
1.32968	0.898767	-1.036427	-3.698634	0.652571	0.974138
2.435739	2.862832	-0.132004	-1.760028	1.014289	-3.697436

Isomer 41			Isomer 42		
X	Y	Z	X	Y	Z
-1.617532	-2.791762	0.962106	-0.289875	1.874478	-3.314151
0.677597	-3.939557	0.562644	0.839624	3.430922	-1.732805
-2.453648	-1.870289	-1.349850	2.97331	1.962939	-1.870578
2.378387	-2.966182	2.211081	1.701796	0.453769	-3.530097
-0.529997	-2.376282	-3.211762	-1.711895	3.033164	-1.624721
1.109989	0.183485	-0.333686	2.156312	2.975778	0.396317
3.361886	0.200071	-0.947318	0.89563	1.010133	-1.155227
-0.706731	0.129092	-2.037613	-0.559678	-0.565193	-3.862193
2.448588	-2.158526	-0.248792	-3.523958	-1.753057	1.649624
1.693680	-1.326753	-2.528941	-2.282064	-2.029909	-2.622684
-2.395288	-0.830685	-3.650154	-1.419617	1.116896	-0.153692
-0.101294	2.636200	-2.653865	-2.237443	0.593323	-2.507431
1.267558	-3.746025	-1.920874	-0.291093	3.261578	0.467615
0.001215	-0.058900	-4.287854	-2.718439	3.074064	0.827758
-0.054892	-1.994464	-0.868395	-1.290847	1.754133	2.431897
2.822353	-0.533639	1.668872	-3.293848	0.674698	1.485192
1.759495	1.042878	-2.815495	-3.155866	-0.751018	-0.621146
2.806194	1.771440	1.051593	-3.788889	1.647454	-0.866984
-1.167325	-4.083312	-1.276982	0.799726	3.097458	2.689606
-3.103467	0.545894	-1.591400	-1.591762	-3.022113	2.411279
-2.055652	1.583514	-3.626024	-1.14096	-0.969879	1.052272
-1.308478	-1.063911	2.913368	-2.188241	-2.970646	-0.184079
-0.019823	-3.277607	2.988221	0.121694	-3.244543	0.628767
-1.601317	-0.280391	0.483362	2.108258	-3.069878	-0.943594
-0.642241	1.924481	-0.189837	-0.293345	-3.485818	-1.805604
-0.215076	4.265778	-0.743612	1.266711	-1.968566	-3.015186
1.771156	2.652668	-0.978036	-0.513214	-1.053933	-1.337066
2.087677	1.410675	3.465829	2.330397	-3.034377	1.697776
0.557368	-1.566485	1.406547	1.395155	-1.113711	0.315249
1.312617	-1.076742	3.652938	0.488976	-1.683115	2.760447
-1.556395	3.575595	1.352623	0.68022	0.65075	3.682062
-0.174349	2.905529	3.377872	2.887641	-0.656339	-1.697681
-0.237313	0.688167	4.385290	3.867356	-1.653804	0.363885
-3.123792	1.808351	0.497617	3.203758	0.748387	0.335934
0.360459	0.876373	1.939857	2.688822	-0.613861	2.427045
-1.976368	1.338546	2.598943	0.779479	1.009719	1.314201
-2.337563	3.029031	-1.548531	2.869385	1.819369	2.580201
0.962321	3.373742	1.290256	-1.763213	-0.549249	3.327785

Isomer 43			Isomer 44		
X	Y	Z	X	Y	Z
0.806352	-2.270979	3.120037	2.587283	-1.823621	1.867562
-0.920445	-0.518232	3.885078	0.602822	-0.19742	2.022429
1.995561	2.496556	2.756128	2.401515	-0.033666	3.674909
2.900453	-1.596634	1.900417	0.005468	0.491787	4.281315
1.503803	0.073758	3.060449	-3.041726	1.278006	0.58269
-0.364594	1.701835	2.805851	-1.910873	0.204712	2.668148
-2.06608	3.262535	1.82266	0.764741	-1.982983	3.664921
1.30821	-1.198698	-0.123888	1.035711	2.353811	-2.116394
-0.513683	-0.735636	1.534665	3.175112	1.385687	-1.127803
-2.650369	-1.709413	2.357513	2.967611	-1.085556	-0.589376
-0.877101	-3.316305	1.671347	-0.432111	3.79012	-0.634038
1.556969	-3.47183	1.056306	2.889485	0.692357	1.296681
-2.639083	0.83365	2.450061	1.752896	2.825247	0.37152
1.156883	1.153195	0.77965	0.15772	4.127983	1.822323
2.713995	3.144538	0.495066	-1.144816	2.540471	3.343602
0.259892	3.49487	1.10715	-0.53307	1.901882	1.07874
3.469201	0.788959	1.444021	1.340999	2.119215	2.821558
3.78117	-1.035337	-0.358492	-2.220711	3.596073	1.168119
2.360405	-2.971723	-1.342671	-0.860168	-3.192242	-2.743053
0.872279	2.903675	-1.208076	0.988809	0.524822	-0.403593
0.471826	0.51854	-1.695009	2.565704	-3.514217	-0.023303
-1.224932	1.318432	0.355387	1.54322	-2.57584	-2.160533
1.962052	1.848554	-3.195822	0.200806	-4.191976	-0.66512
2.915124	1.198117	-0.999714	0.677895	-1.96283	0.163808
2.576228	-0.670627	-2.571061	0.856505	-3.72306	1.847131
0.714503	0.017795	-4.20839	-1.807045	-2.642963	-0.467831
-1.577341	3.360245	-0.847795	-1.222572	-0.359622	0.340475
-1.512169	-0.456616	-3.316862	-3.332161	-1.234999	1.050433
-0.667828	2.083276	-3.109847	-1.156955	-2.216014	2.027456
0.459266	-1.926611	-2.643129	-0.427729	-0.926498	-1.921014
-0.157099	-3.339305	-0.685919	0.39575	-1.503509	-4.155623
-2.650564	-2.780099	0.003383	1.847788	-0.056793	-2.703243
-3.594983	2.1146	0.315009	-0.362382	0.893828	-3.664553
-3.522569	-1.183897	-1.922697	-2.056885	-1.076773	-3.715665
-2.536984	1.12688	-1.723782	-1.134386	3.227802	-3.023625
-1.83426	-2.88765	-2.470027	-1.351558	1.554895	-1.307142
-1.209257	-1.025568	-0.875144	-2.785393	1.265048	-3.262843
-3.264832	-0.34485	0.378153	-2.977299	-0.47316	-1.409066

Isomer 45			Isomer 46		
X	Y	Z	X	Y	Z
-2.172917	-2.852479	1.468358	0.839069	-1.364433	-0.774209
-3.49431	-1.371752	-0.14738	0.617186	-3.981947	-1.055018
-0.811123	-0.785099	1.295794	2.675714	-2.732951	-0.106254
-1.596512	-2.790708	-0.948117	-0.689883	-2.37088	-2.542757
-3.084508	-0.633285	2.271099	-1.824897	-0.4495	-3.749006
-2.832506	-1.56488	-2.709716	-0.607409	1.671962	-3.757926
-3.150807	1.034902	0.429957	0.702112	-0.497623	-3.447698
-1.480138	-0.223793	-1.057682	1.834035	-2.638555	-2.693568
-1.486897	0.61493	-3.396617	3.187415	-0.29913	0.395992
-1.926834	2.254974	-1.560131	3.699056	1.636018	-1.246144
-3.704583	0.574383	-1.93859	1.724655	1.710874	-2.923184
-1.034785	-0.320467	3.721899	2.783411	-0.473686	-2.055373
-2.433137	3.398619	0.576701	1.300372	1.162822	-0.561137
-2.104598	1.708446	2.593303	2.034876	3.504542	-1.214992
-0.366027	-1.571475	-2.934021	3.125079	0.5882	2.681538
0.464964	-3.698111	-1.977986	2.378685	-1.866274	2.325314
0.100414	-3.705516	0.650666	0.414325	-2.984107	1.175462
-0.130946	-2.537152	2.919403	0.882262	-0.184051	1.532516
1.596514	2.790711	0.94812	-1.711993	-3.346033	-0.493654
-0.651616	1.612343	0.573754	1.198691	-0.10237	4.019301
3.494312	1.371753	0.14738	-1.211528	3.197446	2.421395
0.366026	1.571474	2.934022	-1.044669	0.809514	3.274476
2.832506	1.564878	2.709718	1.043825	2.067334	2.700134
0.651618	-1.61234	-0.573755	-1.296453	-1.158538	0.561694
3.150809	-1.034902	-0.429957	-3.694386	-2.003034	0.490633
3.704585	-0.574384	1.93859	-2.820962	-0.723424	2.446792
2.433135	-3.398617	-0.576702	-0.47219	-1.618449	3.058089
1.486895	-0.614931	3.396617	-2.035461	-3.143702	1.973478
1.926834	-2.254972	1.560131	-3.134959	1.715831	2.13019
1.48014	0.223795	1.057682	-2.778617	-1.308952	-1.65962
0.130944	2.53715	-2.919405	-2.67145	1.614031	-2.198989
-0.100414	3.705518	-0.650667	-3.193531	0.486728	0.012486
1.034783	0.320463	-3.721899	-0.887735	1.325108	0.788615
0.811124	0.7851	-1.295793	-0.828278	0.238075	-1.557082
2.104597	-1.708448	-2.593303	-0.420188	2.779779	-1.576509
3.084508	0.633282	-2.271101	-2.409591	2.979884	-0.007545
-0.464967	3.698111	1.977987	0.480341	3.403668	0.660226
2.172916	2.852477	-1.468358	2.813075	2.355826	0.972337

Isomer 47			Isomer 48		
X	Y	Z	X	Y	Z
1.196400	-2.014938	3.258669	3.470329	1.702127	-0.954012
0.953449	0.379012	4.230741	3.818205	0.296187	1.090425
3.059008	-1.891305	1.375020	2.113067	2.15174	1.119354
3.144048	-0.184167	3.186770	-2.197015	3.166368	1.001165
3.025105	-1.104158	-1.150461	0.588101	2.416877	3.107601
1.146971	-0.122120	1.801136	-0.407355	1.413111	1.04084
1.341501	-2.616572	-2.483246	2.37059	0.65549	3.129144
1.133165	-3.203385	-2.730199	1.673312	3.477718	-0.976526
3.168923	-0.008682	-0.622677	0.238667	3.834525	1.046357
0.399069	-0.991435	-1.853679	-1.848196	1.749611	3.062815
0.951050	-0.421740	0.503529	1.051428	1.051094	-1.015874
2.945556	-1.303784	1.552428	2.480432	0.676382	-3.038948
0.798766	-2.656477	1.667335	-3.179346	2.147692	-1.066919
2.098446	-2.390623	-0.583049	0.684022	2.451075	-3.061347
1.396517	-3.820823	1.550992	-0.784278	2.931041	-1.071279
0.004440	-3.966302	-0.644291	-2.953706	0.762685	1.025623
1.064432	-0.429652	3.020297	-1.733296	1.788937	-3.106465
2.641599	-3.576568	-0.589434	-1.404928	0.378291	-1.062605
0.994782	-1.895185	0.031187	-2.480432	-0.676382	3.038948
3.263462	0.661198	0.776072	-0.684022	-2.451075	3.061347
1.948765	2.064375	2.597899	1.404928	-0.378291	1.062605
0.213227	-1.443340	-4.232554	-2.113067	-2.15174	-1.119354
1.556839	-0.031877	-3.044138	-1.051428	-1.051094	1.015874
0.826845	0.898289	-3.649872	-0.055992	-0.016477	3.217961
2.516629	-1.017108	-2.959913	-1.673312	-3.477718	0.976526
1.488984	3.245861	-2.701403	-3.470329	-1.702127	0.954012
0.882305	2.386654	-2.279344	0.784278	-2.931041	1.071279
1.109735	1.336861	-1.246588	-3.818205	-0.296187	-1.090425
3.141342	1.383854	-2.687278	2.197015	-3.166368	-1.001165
0.448589	3.787212	-0.504913	1.733296	-1.788937	3.106465
1.239489	0.549293	-0.653938	-0.238667	-3.834525	-1.046357
1.937336	2.810195	0.081688	-0.588101	-2.416877	-3.107601
1.852467	3.601597	1.618902	0.407355	-1.413111	-1.04084
0.588766	2.133270	3.350457	-2.37059	-0.65549	-3.129144
2.533206	1.205208	1.606412	3.179346	-2.147692	1.066919
0.158708	1.876801	1.014824	1.848196	-1.749611	-3.062815
2.882931	2.748351	-0.488998	2.953706	-0.762685	-1.025623
0.588821	4.022207	1.881616	0.055992	0.016477	-3.217961

Isomer 49			Isomer 50		
X	Y	Z	X	Y	Z
-0.562758	1.609027	0.608502	-1.593841	1.492479	-0.038794
-0.930881	3.921724	-1.59042	-2.263838	3.82706	-0.657709
-2.145768	1.756757	-1.490197	-3.470523	0.013919	0.871584
1.60056	0.437879	0.271898	-1.117183	2.471805	-2.415694
-2.287767	3.37123	0.438588	-3.44204	1.710572	-1.595334
2.213966	2.622274	1.100913	-2.825358	-0.696934	-1.339484
0.157724	4.015384	0.660382	-0.905332	-0.853417	0.423068
3.831818	0.575246	1.171209	-0.567819	0.9318	2.380573
1.163875	2.675777	-1.199009	1.619716	0.8802	3.591491
0.187081	0.428772	-1.736137	-2.886735	1.444651	2.879129
1.939694	1.611236	-3.322058	0.866827	0.961425	0.139593
3.272765	1.180296	-1.267289	1.141769	2.705476	1.920421
2.173046	-0.754638	-2.601579	-3.708961	2.513016	0.829386
0.342539	-0.112965	-4.19563	2.617599	2.71987	-0.113033
-1.846798	-0.253898	-2.999236	-1.383321	3.2144	1.712034
-0.512127	2.150148	-3.313274	3.026755	0.847573	1.536384
-0.154803	-2.045524	-2.635755	-0.026819	-0.96252	3.961856
-3.817222	-0.161643	-1.582328	0.182705	3.345895	-0.368075
-2.369644	-2.424072	-1.502502	-2.213403	-0.969379	2.70607
-2.460658	-0.41689	2.246636	-1.135464	-2.392107	-1.734828
-2.131955	2.014052	2.548893	3.439938	-1.892051	-1.87279
-1.179463	-2.150061	3.426544	-0.406336	0.101646	-1.890176
-0.572755	0.475124	3.781692	2.457286	-3.627889	-0.445275
0.269679	2.653488	2.761397	1.17002	-2.921137	-2.577719
1.833943	0.74147	2.810045	-0.38277	-1.373198	-3.877919
2.001949	-3.14796	-2.027469	0.031009	-3.481672	0.085379
0.794266	-1.744067	-0.356054	1.298928	-1.401822	-0.689747
2.474207	-1.491571	1.761182	3.386196	-1.534785	0.69046
-1.665466	-2.62692	0.961723	1.81042	-0.437853	-3.058003
3.368231	-1.342691	-0.545521	1.39559	1.949616	-2.17242
1.153319	-1.459003	3.84777	0.114197	1.07675	-4.145024
2.409484	-3.557694	0.329413	3.096505	0.425641	-1.003226
0.560814	-3.152251	1.888197	1.852369	-3.191105	1.921726
-0.038839	-0.717436	1.739427	1.13158	-0.881681	1.824313
-3.815993	-1.486268	0.465518	3.060223	-1.167986	3.17525
-1.612563	-0.45943	-0.299092	-2.237922	0.448301	-3.470355
-3.421829	1.109761	0.56801	-2.522502	-2.541391	0.5211
-0.221673	-3.844667	-0.72439	-0.609463	-2.755169	2.295788

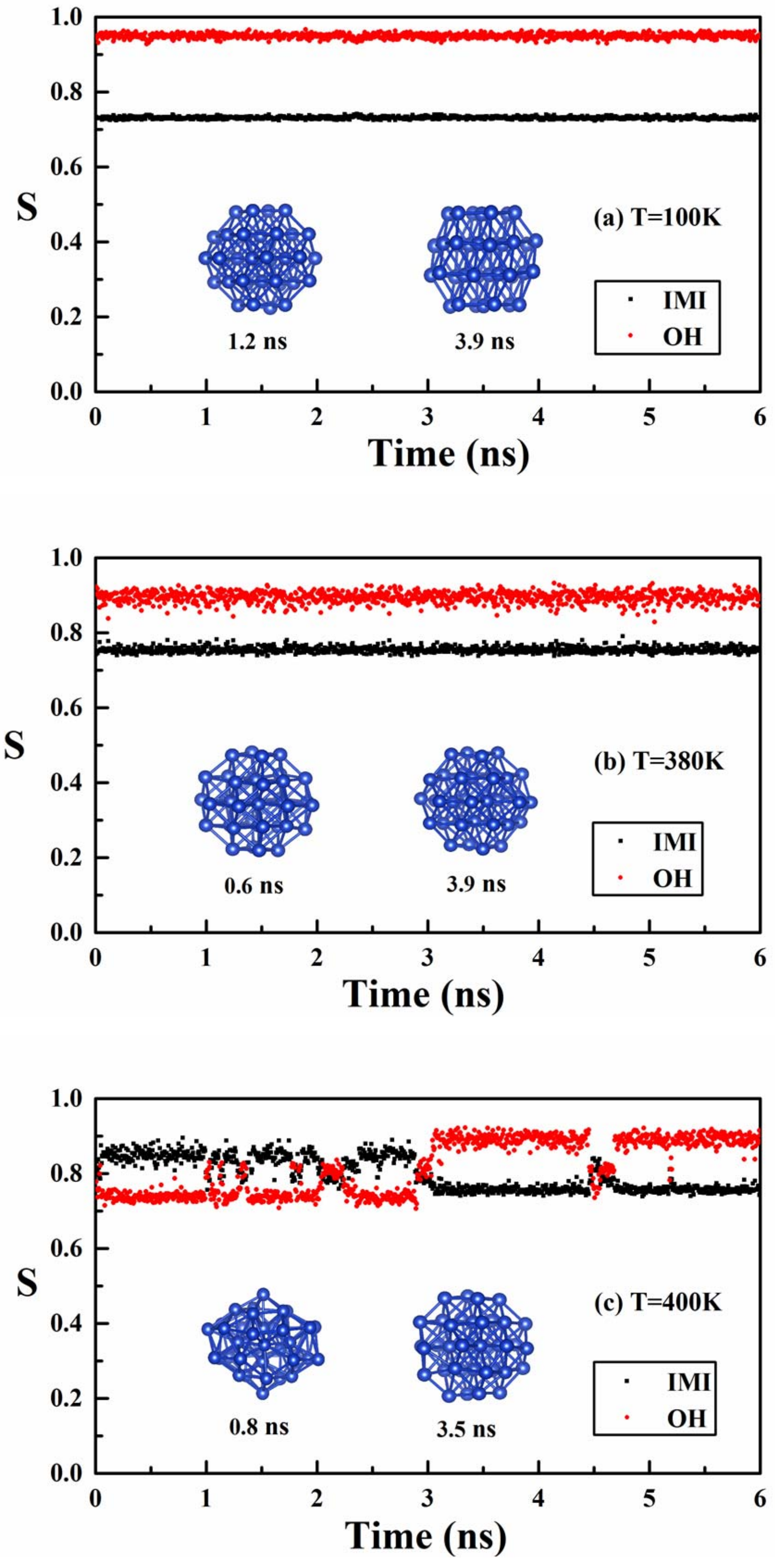

1.0
0.8
0.6
0.4
0.2
0.0
S
(a) T=100K
1.2 ns
3.9 ns
IMI
OH
0
1
2
3
4
5
6
Time (ns)
(b) T=380K
0.6 ns
3.9 ns
(c) T=400K
0.8 ns
3.5 ns

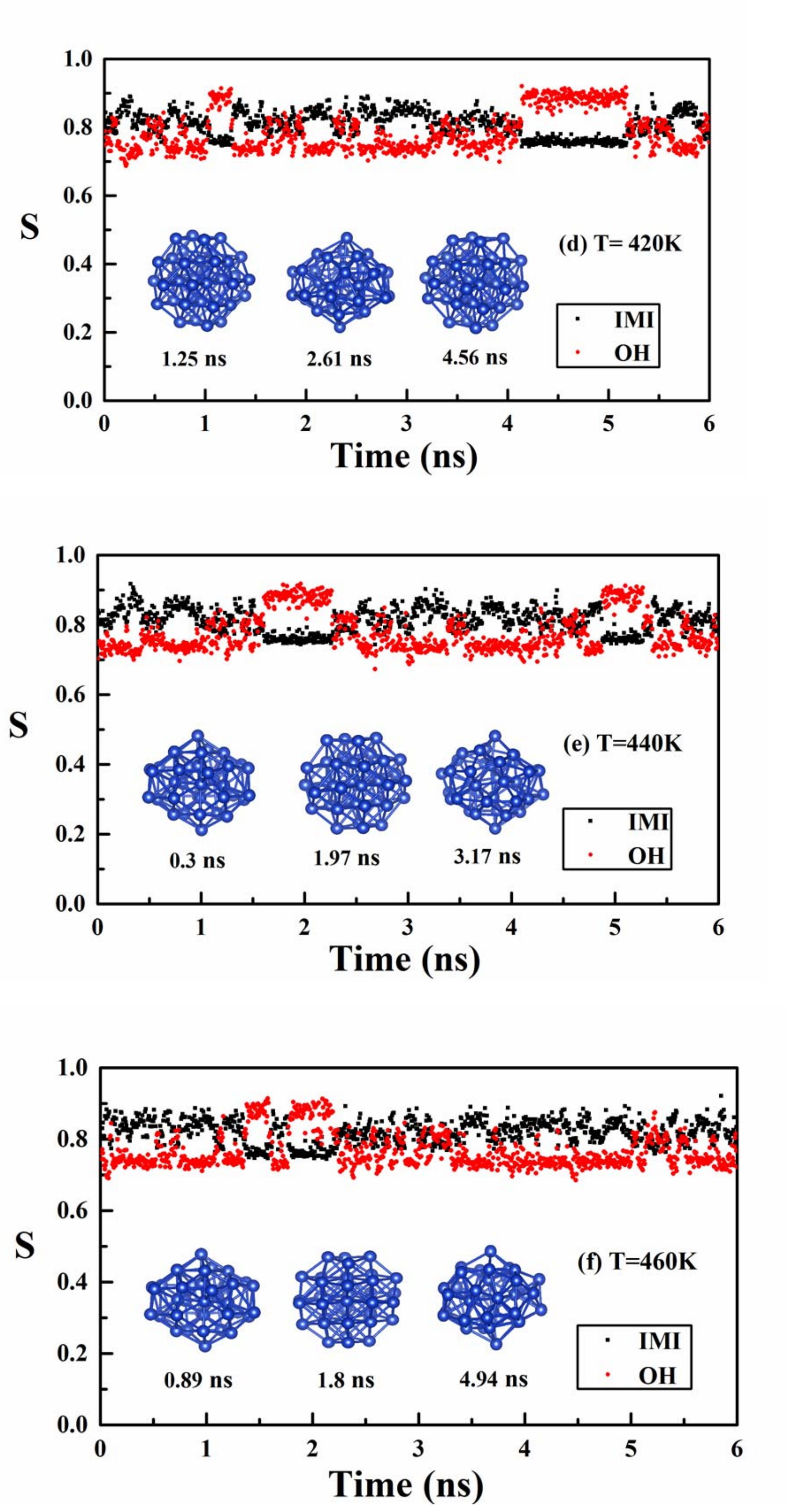

(d) T= 420K
1.25 ns
2.61 ns
4.56 ns
IMI
OH
S
Time (ns)
(e) T=440K
0.3 ns
1.97 ns
3.17 ns
IMI
OH
S
Time (ns)
(f) T=460K
0.89 ns
1.8 ns
4.94 ns
IMI
OH
S
Time (ns)

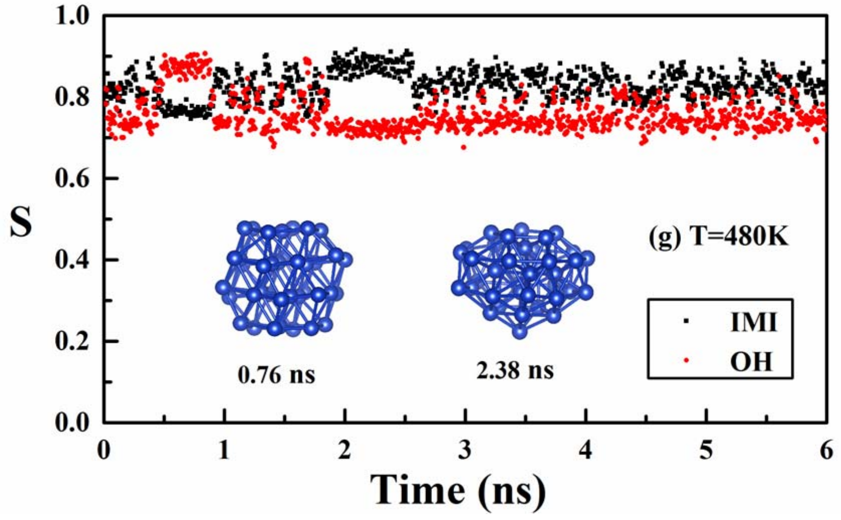

1.0
0.8
0.6
0.4
0.2
0.0
S
0
1
2
3
4
5
6
Time (ns)
(g) T=480K
0.76 ns
2.38 ns
IMI
OH

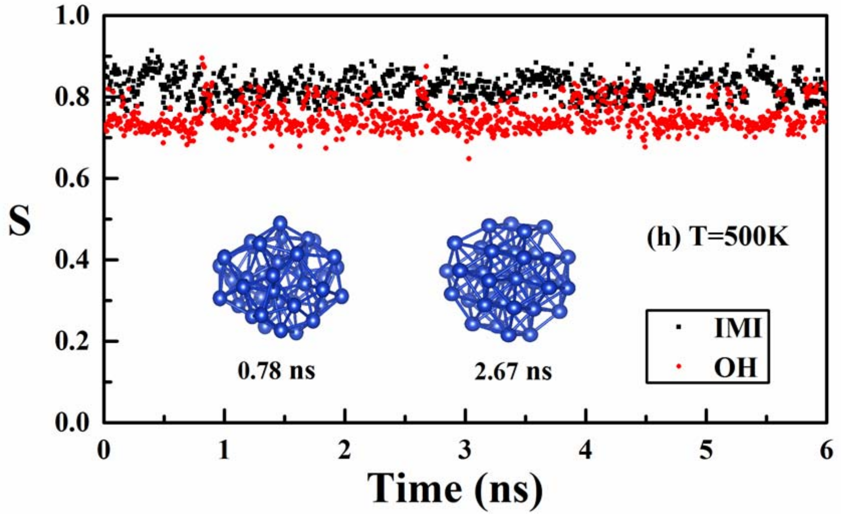

1.0
0.8
0.6
0.4
0.2
0.0
S
0
1
2
3
4
5
6
Time (ns)
(h) T=500K
0.78 ns
2.67 ns
IMI
OH

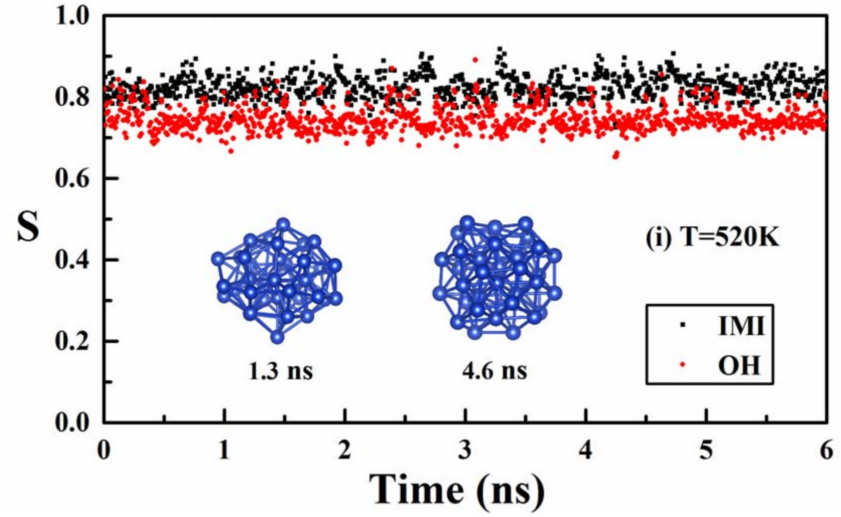

1.0
0.8
0.6
0.4
0.2
0.0
S
0
1
2
3
4
5
6
Time (ns)
(i) T=520K
1.3 ns
4.6 ns
IMI
OH

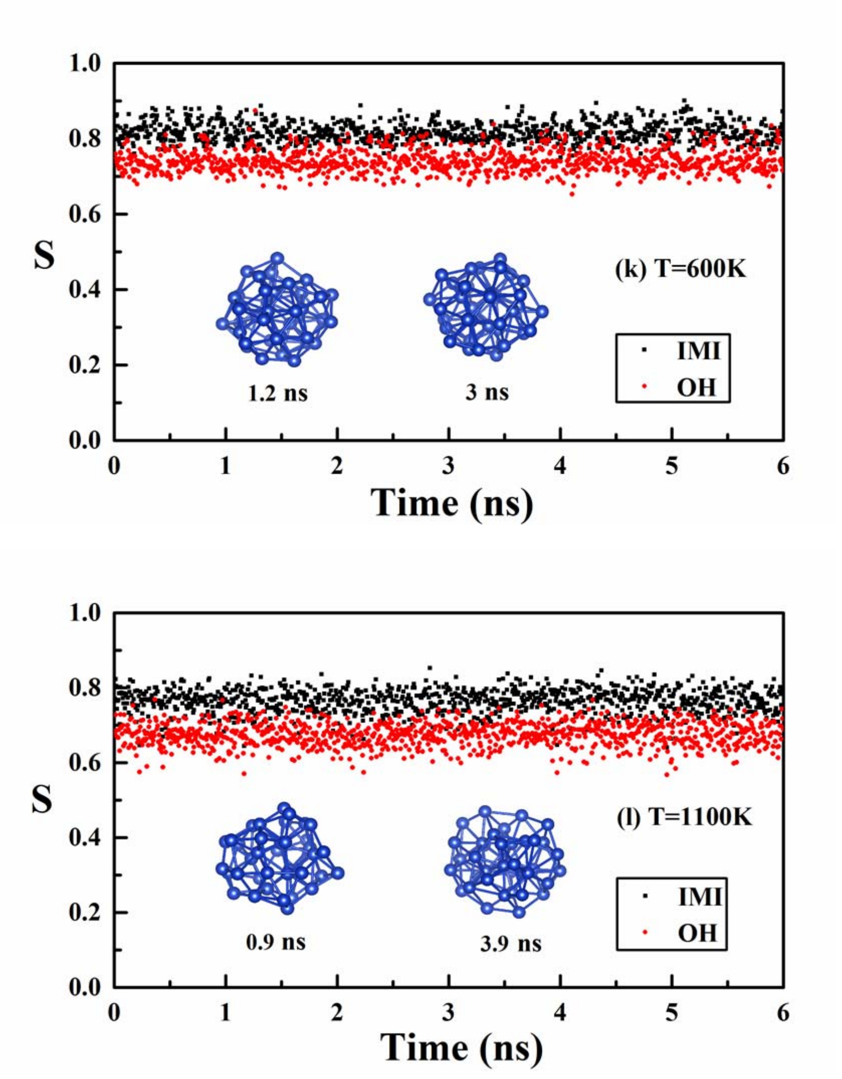

Figure S1 The snapshots of Cu_{38} at different temperatures. At 380 K and before, Cu_{38} remains in a solid-like state; From 400 K to 520 K, as corresponding the pre-melting stage, the IMI-like and OH-like structures dominate the dynamical process; At temperatures higher than 540 K, Cu_{38} can undergo many different structures and the cluster remains in a liquid-like state.
